# Mineralogical and Elemental Variability of Canine Dental Calculi Reveals an Age-Associated Calcite–Apatite Continuum

**DOI:** 10.1021/acsomega.6c07170

**Published:** 2026-10-09

**Authors:** Marcello Campione, Andrea Lucotti, Matteo Tommasini, Claudio Pezzoli, Valerio Cerantola, Giancarlo Capitani

**Affiliations:** † Department of Earth and Environmental Sciences, University of Milano - Bicocca, 20126 Milano, Italy; ‡ Department of Chemistry, Materials, and Chemical Engineering, 18981 Politecnico di Milano, 20133 Milano, Italy; § Clinica Veterinaria Bicocca, Viale Fulvio Testi 177, 20162 Milano, Italy

## Abstract

Dental calculus is a mineralized oral biofilm widely occurring in domestic dogs, yet its mineralogical variability and chemical evolution remain insufficiently defined. Here, we investigate dental calculus collected as biological waste material during routine professional dental cleaning from twenty-eight indoor pet dogs of different breeds, ages, and clinical backgrounds. X-ray powder diffraction, vibrational spectroscopy, and elemental analysis were combined to determine the crystalline phase assemblage, phosphate and carbonate spectral signatures, and major-element composition of the deposits. The crystalline fraction is dominated by hydroxyapatite and magnesian-calcite, which occur over a broad compositional range from calcite-rich to apatite-rich deposits. Raman analysis of selected apatite-rich samples further indicates the presence of structurally disordered phosphate contributions compatible with amorphous calcium phosphate, while quantitative phase analysis of a representative apatite-rich sample reveals a substantial amorphous fraction. Elemental data show that phosphorus increases monotonically with the crystalline hydroxyapatite fraction, whereas calcium and magnesium remain comparatively constant across the sample set. The Ca/P ratio is markedly higher than that of stoichiometric hydroxyapatite and decreases nonlinearly with increasing hydroxyapatite fraction, supporting the presence of calcium-bearing nonapatitic components and confirming major-element chemistry as an independent descriptor of the calcite–apatite balance. Correlation and exploratory multivariate analyses indicate that hydroxyapatite enrichment is moderately associated with dog age, ablated calculus mass, and estimated accumulation time, defining an age- and accumulation-associated mineralization continuum rather than a fixed calculus composition. These results provide a mineralogical and chemical framework for canine dental calculus as a heterogeneous biomineral system and suggest that its composition records coupled processes of biofilm mineralization, deposit accumulation, and mineral maturation. The study is cross-sectional and does not validate diagnostic use, but establishes a basis for future controlled and longitudinal investigations of dental calculus mineralization in canine oral health.

## Graphical abstract

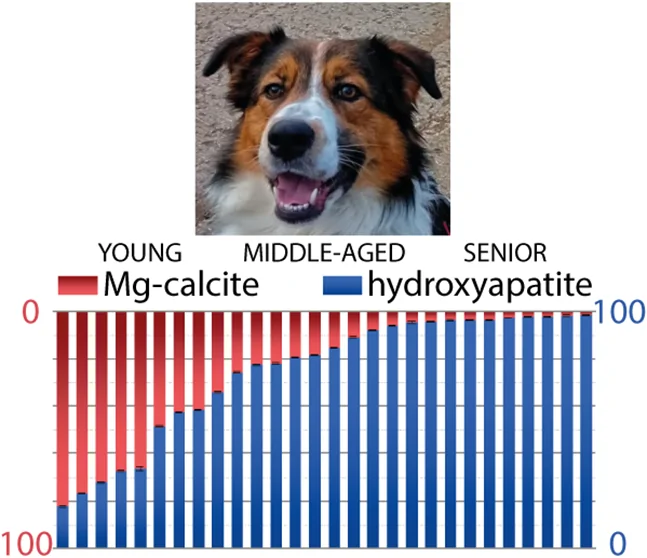

## 1 Introduction

Dental plaque is one of the most common and extensively investigated biofilms in animals. Its development begins with the adsorption of salivary proteins onto the tooth surface and the adhesion of pioneer microorganisms, followed by interbacterial coadhesion and microbial succession toward a more complex community.^1^ Plaque mineralization with formation of dental calculi (DC, also referred to as tartar), occurs when the local fluid becomes supersaturated with respect to calcium-phosphate salts, a condition strongly influenced by pH and alkalinizing bacterial metabolism.^2^ Mineral nucleation may occur heterogeneously on bacterial cell surfaces, membrane phospholipids and proteolipids, extracellular-matrix components, membrane-derived vesicles, and cellular debris. Salivary proteins, pyrophosphate, magnesium, and other promoters or inhibitors can further regulate nucleation, crystal growth, and phase selection.^3,4^ Mechanical removal of dental biofilm is common practice in humans to prevent acids and toxins produced by biofilm bacteria from attacking enamel, damaging periodontal tissues, and inducing gingival inflammation, with eventual tooth loss. This prevents the formation of significant amounts of DC in humans, whereas, in pets, e.g., domestic dogs, regular dental cleaning is impractical and formation of considerable amounts of DC is rather common.

The global domestic canine population is continuously growing, reaching today an estimated number of half a billion, and the high prevalence of oral disorders in pet dogs has been well assessed in recent studies.^5,6^ The most frequent oral disorder is periodontal disease (PD),^7^ with DC being an inherent component of the former.^8^ It is generally accepted that the inflammation and resulting tissue damage is due to an improperly regulated immune response to bacterial infection.^6,9^ Given the potential link between DC and systemic health, scientific efforts should first address their physical-chemical characteristics, and then their relationship with canine health status.

Several calcium-phosphate phases have been identified in human DC, including brushite or dicalcium phosphate dihydrate, octacalcium phosphate, Mg-containing whitlockite, and carbonate-containing hydroxyapatite.^10^ Brushite is predominantly associated with early supragingival deposits, whereas octacalcium phosphate, hydroxyapatite, and whitlockite have been reported in both supragingival and subgingival calculus. Current models propose that calculus mineralization may initially involve amorphous or poorly ordered calcium phosphate and kinetically favored precursor phases such as brushite and octacalcium phosphate, followed by hydrolysis and maturation toward less soluble hydroxyapatite and whitlockite.^11^ This evolution should not be regarded as a unique linear pathway, because local pH, fluid composition, magnesium, fluoride, and macromolecular inhibitors can favor or stabilize different phases.^3,4^

The most practical way to analyze DC is to determine their mineral composition. LeGeros & Shannon pioneered the investigation of the crystalline composition of DC in dogs,^10^ analyzing samples from approximately ten individuals. They illustrated by X-ray methods that the main components are Ca-carbonate in the form of calcite, and Ca-phosphate in the form of apatite. The calcite/apatite ratio was observed to vary from dog to dog because of variation of the carbonate/phosphate concentration in the dog saliva. This ratio was higher in the innermost layer of the calculus sample compared to the outermost layer, suggesting that calcite is prone to hydrolysis to apatite in the presence of phosphate in dog saliva. The predominance of calcite mixed with small amounts of apatite is a consequence of both the conditions of high pH and high carbonate concentrations in the dog saliva, together with the higher pH of resting plaque induced by the proteolytic activity.^12^ Indeed, the dog saliva is supersaturated with respect to calcium carbonate, and phosphate anions select the precipitation of Ca-carbonates in the form of the calcite polymorph.

Later, LeGeros et al. evaluated the efficacy of scanning electron microscopy (SEM) and electron probe microanalysis to reveal the solid components of human and animal DC, including dogs.^13^ In that study, quantitative analysis of Ca, Mg and the Ca/P ratio are reported, showing that the Ca/P molar ratio of animal calculi sets above 2.5 (1.3–1.7 in human) and the Mg content is not correlated with the Ca/P ratio. Ratios above 15 were attributed to regions exclusively composed by calcite.

Recently, Chiba-Ohkuma et al. provided a multimethodological analysis of seven, aged, indoor dogs (>15 y), confirming the higher Ca/P ratio in dog calculi with respect to human ones and also a larger fraction of organic matter, attributed to the absence of amylase in dog saliva.^14^ X-ray diffraction analysis suggested a mixture of calcite and carbonate-bearing hydroxyapatite.

Nakashima et al., starting from the evidence of ease of detachment of dog calculi as compared to human ones, analyzed the morphology and composition of the enamel/calculus interface.^15^ They showed the presence of a smooth surface and a 100 μm-wide gap demonstrating the poor adhesion. Microanalysis showed that the surface in contact with the enamel is characterized by a reduced content of Ca and P, which is not sufficient to establish if it is calcite-rich or phosphate-rich. In relation to the studies reported by Borah et al. −establishing that the inorganic components of canine saliva constrain the precipitation of carbonated hydroxyapatite −the authors claim that further studies are needed to bridge the mineralogical gap in canine calculus composition, in particular due to the pivotal role of Ca-salts for calculus formation.^16^

The ionized form of Ca is the most important in biochemical reactions. More severe PD staging are generally associated with lower serum Ca^2+^ values.^17^ Interestingly, statistically significant correlations were observed between age, serum Ca^2+^ levels, and PD stage,^18^ while no such correlations were established with sex or weight. PD manifests approximately in 80–85% of dogs older than 2 y, with the disease affecting nearly all animals older than 5 y, with older dogs exhibiting more advanced stages of the disease, since PD stage becomes worse as the patient grows older.

In this work, we combine quantitative mineralogical analysis, major-element determination, and exploratory statistical analysis on DC collected from a population of 28 dogs. The results show that canine DC is not characterized by a fixed mineralogical composition, but spans a broad calcite–apatite continuum. This compositional variability defines a mineralogical maturation/accumulation trajectory, ranging from calcite-rich deposits, interpreted as comparatively early stage or less mature mineralization products, to apatite-rich deposits, representative of a more advanced mineralization state.

## 2 Materials and Methods

### 2.1 Sampling of Dental Calculi

DC were obtained as biological waste material during routine professional dental cleaning procedures using an ultrasonic scaler under general anesthesia performed by a licensed veterinarian (Clinica Veterinaria Bicocca, Milano, Italy) for oral hygiene and disease prevention purposes of 28 indoor dogs of various breeds. The collected material included both supragingival and subgingival calculus. Because routine ultrasonic scaling progressively detaches material from both locations, reliable separation of the two fractions during the clinical procedure was not practically feasible. They were therefore analyzed as bulk calculus. Accordingly, the present study addresses the overall mineralogical composition of the material removed during professional dental cleaning and does not resolve site-specific differences between supragingival and subgingival deposits. Dogs were included opportunistically when professional dental cleaning was clinically indicated by the veterinarian and when owner consent for anonymized scientific use of the removed material was obtained. No selection was made on the basis of breed, sex, reproductive status or expected mineralogical composition. No procedures beyond standard veterinary care were carried out for research purposes. The study protocol was evaluated by the Organismo Preposto al Benessere Animale (OPBA; Animal Welfare Body, AWB) of Clinica Veterinaria Bicocca (Milano, Italy), which confirmed that formal ethical approval was not required under Legislative Decree 26/2014 and Directive 2010/63/EU, as the research involved only biological waste material obtained during routine veterinary dental procedures and no experimental interventions were performed on live animals. The study is reported in accordance with the ARRIVE guidelines (https://arriveguidelines.org). No animals were euthanized for this study. Informed consent was obtained in written form from all dog owners prior to the clinical procedure and sample collection. Owners were informed that part of the material removed during professional cleaning could be anonymized and used for scientific analysis of mineral composition. All samples were handled following GDPR-compliant anonymization protocols to ensure that no identifying information about the dogs or owners could be retrieved.

Clinical information was retrieved from routine veterinary records. A standardized periodontal staging protocol including systematic periodontal probing and intraoral radiography was not performed for research purposes. Therefore, the oral-health score used here (see section 2.5) should be considered an exploratory ordinal descriptor based on reported clinical observations, not a formal periodontal diagnosis. For each dog, the following information was recorded: ID, age, breed, weight, sex and reproductive status, history of previous dental cleanings, feeding habits, reported pathologies and surgical procedures, as well as the main outdoor environments frequented. Calculus samples were weighted (the mass of collected calculi samples varied from 10 to 800 mg) and dried in air for 24 h after extensive rinsing with deionized water. Because collection during routine ultrasonic scaling was not designed to quantitatively recover the entire calculus burden, this variable represents the mass of material collected for analysis rather than the total amount of calculus present in the oral cavity. It was therefore used as an operational proxy for calculus accumulation and should not be interpreted as an exact measure of total calculus burden. Sample codes (e.g., MNU, MLU, GMA, etc.) refer to anonymized individual dog identifiers. Collected information related to the investigated dogs is arranged in Table S1.

Samples were powdered in a mortar and stored in desiccators. Subsequent analyses were performed using the following methods.

### 2.2 X-Ray Powder Diffraction (XRPD)

An X’Pert Pro X-ray diffractometer (Malvern Panalytical) with Bragg–Brentano geometry (specular scans) and Cu target (λ = 1.54 Å) as X-ray source was used to collect diffraction patterns on the powdered samples to determine mineral composition and relative occurrence. Given the low amount of sample available for some dogs and to adopt a common methodological approach for all of them allowing for a consistent comparison, data collection was performed using a low-background planar sample holder, enabling the measurement of very small sample amounts. However, while delivering a data set suitable for reliable determination of calcite and apatite proportions, this technique could not be employed to determine the amorphous content accompanying the crystalline phases, which requires the mixing with a known amount of an internal standard (corundum), making this mixed sample no longer suitable for subsequent analyses. On the contrary, for a representative voluminous samples (LLI, mass = 320 mg), data collection was also performed with corundum internal standard, enabling quantitative phase analysis (QPA) also of the amorphous fraction (see Supporting Information). QPA was undertaken by Rietveld refinement using Profex v. 5.6.1.^19^

### 2.3 Raman Spectroscopy

The micro-Raman measurements were carried out with the HR800 Horiba Jobyn-Yvon instrument using a 50× objective (0.75 NA, laser spot size about 1 μm) and the 405 nm excitation provided by a solid-state laser produced by Integrated Optics. Before measurements, the sample powder was gently pressed on a microscope glass slide. For each different point on the powder sample, the laser focusing procedure was repeated before collecting the Raman spectra. The different points on the sample were randomly selected within a zone of a few millimeters in diameter. For each powder sample, we collected several spectra by focusing the laser on different points of the available material. We observed negligible variations from point to point; thus, the reported spectra can be considered representative of each sample. The laser power incident on the sample was kept below 10 mW for all measurements to avoid photodamage.

### 2.4 Quantitative Analysis of Major Elements

Powder samples were dissolved in a mixture of 4 mL HNO_3_ 65%, 4 mL HCl 37%, and 2 mL H_2_O_2_ 40% by the aid of a microwave digestor (MV 5000, Anton Paar) undertaking a thermal treatment at 200 °C for 10 min. The concentration of Ca, Mg, and P contained in the dissolved calculi were determined by using an Inductively Coupled Plasma-Optical Emission Spectroscopy (ICP-OES) Optima 7000 DV (PerkinElmer, Italy) system.

### 2.5 Statistical Analysis

A unified data set was constructed by matching each DC sample with the corresponding dog record using the anonymized sample ID. The mineralogical descriptor used for statistical analysis was the hydroxyapatite fraction obtained by Rietveld refinement with Profex, hereafter indicated as HA%. The calcite fraction was calculated as CA% = 100 – HA%, considering only the crystalline fraction resolved by XRPD. Additional quantitative variables included dog age at ablation, body weight, ablated calculus mass, time elapsed since the previous dental cleaning, and the elemental concentrations of Ca, Mg and P determined by ICP-OES. The Ca/Mg and Ca/P weight ratios were calculated from the corresponding elemental concentrations.

The time elapsed since the previous dental cleaning was defined as the number of months between birth and the sampling ablation for dogs undergoing their first dental cleaning, and as the number of months between the previous dental cleaning and the sampling ablation for dogs that had undergone one or more previous dental cleaning procedures. This variable was therefore used as an estimate of the effective accumulation time of the calculus deposit.

Reported oral conditions were converted into an ordinal oral-health score for exploratory statistical analysis. Dogs with no reported systemic or oral pathology were assigned score 0. Dogs with gingivitis, including minor, modest or moderate gingivitis, were assigned score 1. Dogs with modest or moderate periodontitis were assigned score 2. Dogs with periodontitis were assigned score 3. Dogs with severe periodontitis and/or tooth mobility were assigned score 4. Dogs for which oral-health information was not available were excluded from analyses involving the oral-health score. Systemic conditions, when reported separately from oral conditions, were not included in the oral-health score. Since this score is not a formally validated periodontal stage, its relationship with other parameters (e.g., the fraction of mineral phases) has no predictive significance.

Univariate associations between quantitative variables were evaluated using both Pearson’s correlation coefficient (*r*), to assess linear relationships, and Spearman’s rank correlation coefficient (*ρ*), to assess monotonic relationships less sensitive to nonlinearity and outliers. Simple linear regressions were performed for selected relationships, including HA% as a function of dog age, HA% as a function of ablated calculus mass, ablated calculus mass as a function of time elapsed since the previous dental cleaning, and Ca/P as a function of HA% or its reciprocal transformation. The reciprocal transformation 100/HA% was also considered because the Ca/P ratio is expected to vary nonlinearly with the relative abundance of the phosphate-bearing crystalline phase.

Exploratory multivariate analysis was performed using Python 3 with the pandas, scipy, statsmodels and scikit-learn packages, to evaluate whether the compositional variability of canine DC could be described by coupled age-, accumulation- and composition-related descriptors. Multiple linear regression models were fitted using statsmodels, with HA% as the dependent variable and dog age, time elapsed since the previous dental cleaning, ablated calculus mass and, where appropriate, oral-health score as explanatory variables. Variance inflation factors were calculated to evaluate collinearity among predictors.

Principal component analysis was carried out on standardized variables to identify latent axes of covariation within the data set. A first principal component analysis (PCA) was performed using scikit-learn after standardization of the input variables on the core quantitative variables available for all dogs, namely dog age, time elapsed since the previous dental cleaning, ablated calculus mass and HA%. A second PCA was performed on the subset of samples for which complete chemical data were available, including HA%, 100/HA%, dog age, time elapsed since the previous dental cleaning, ablated calculus mass, Ca%, Mg%, P% and Ca/P. PCA was used as an exploratory tool and not as a predictive model. Scores and loadings were inspected to identify compositional trends, accumulation-related trends and samples deviating from the main age–accumulation–composition axis.

All statistical analyses were performed on the complete 28-dog data set when the variables were available. Analyses involving oral-health score were restricted to dogs with known oral-health information. Statistical significance was assessed at *p* < 0.05. Given the limited cohort size and the exploratory nature of the multivariate analysis, results were interpreted primarily in terms of effect size, direction of association and consistency among variables rather than as definitive causal evidence.

## 3 Results

### 3.1 Mineralogical Characterization of Dental Calculi

An accurate quantitative XRPD measurement performed on a representative and particularly massive sample (LLI) is reported in Figure 1. The full indexation of the diffraction peaks is accomplished by considering the presence of hydroxyapatite (HA, Ca_5_(PO_4_)_3_(OH)) and magnesian calcite [CA, (Ca,Mg)­CO_3_]. Related peak positions, *hkl* indexes and relative intensities of major peaks are reported in Table S2a. The relatively broad peaks of the HA phase indicate its lower degree of crystallinity as compared to CA. Accordingly, besides the two minerals HA and CA, an amorphous phase composes the sample for a fraction as high as 61.4% (Figure S1 and Table S2b).

**Figure 1:**
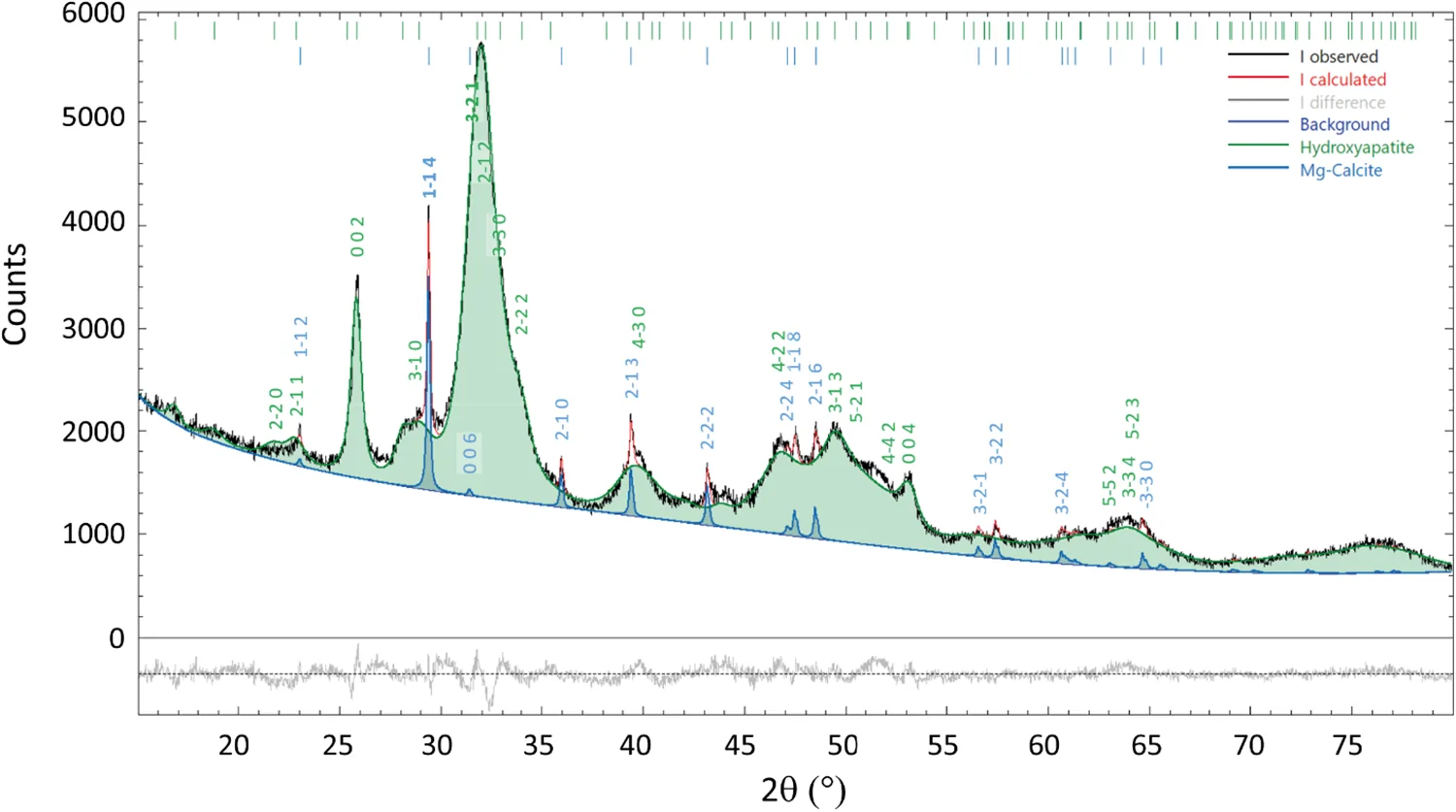
Sample LLI. Experimental (black) vs calculated (red) diffraction pattern and related intensity difference (gray). Major peaks for Mg-Calcite (light blue) and hydroxyapatite (green) are indexed. Most intense peaks, for both phases, are reported in bold: 3 −2 1 for HA and 1 −1 4 for CA. For a detailed list of *hkl* reflections, peak positions and relative intensities, refer to Table S2a.

Magnesian calcite was suggested as the highest matching pattern in the 87% of cases (see Supporting Information). The amount of MgCO_3_ estimated from the refined *a* parameter is between 0.86 and 2.33 mol %, with an average of 1.16 mol % (Table S3).^20^ On this basis, the mineral can be defined a low-Mg calcite.^21^

After establishing that these three phases compose the canine calculi, their relative amount can be estimated by comparing the intensity of the corresponding XRPD reflections via Rietveld refinement.

Figure 2 reports a selection of XRPD patterns – only every other pattern along the series reported to improve readability. The whole series of 28 XRPD patterns acquired on an equal number of different samples is reported in the Supporting Information as Figure S2. As can be observed, the amount of HA and CA spans all the proportions, from almost purely apatitic, to almost purely calcitic (Figure 3), with a distribution of the HA% measured showing an increasing trend (Figure S3). The visual inspection of Figure 2 confirms the reliability of QPA. Actually, the main diagnostic peaks of the two crystalline phases vary consistently along the series, with few exceptions, i.e., PTA, which probably should occupy a lower position (see also PTO in Figure S2). However, it should be noted that both PTA and PTO belong to the HA-richest region characterized by a plateau in Figure 3, where the relative proportions between HA and CA vary within the experimental error (Table S3).

**Figure 2:**
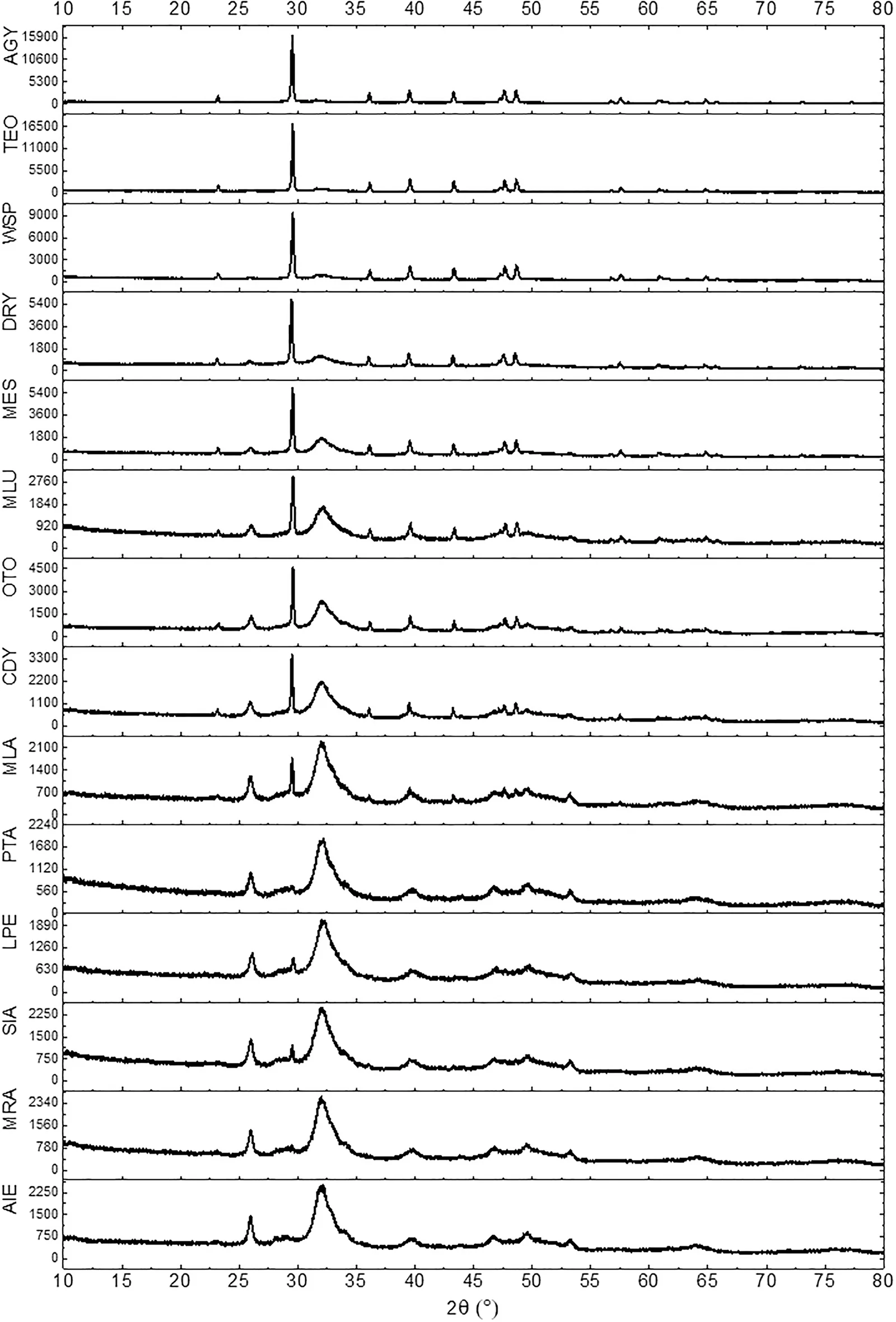
Selected XRPD patterns of DC – only every other pattern along the series has been selected – showing a continuous variation from almost pure HA composition (bottom) through almost pure CA composition (top). The amorphous phase, present in all samples, could not be estimated; however, it can be inferred from the gibbosity present below the main peaks, especially in HA-rich patterns.

**Figure 3:**
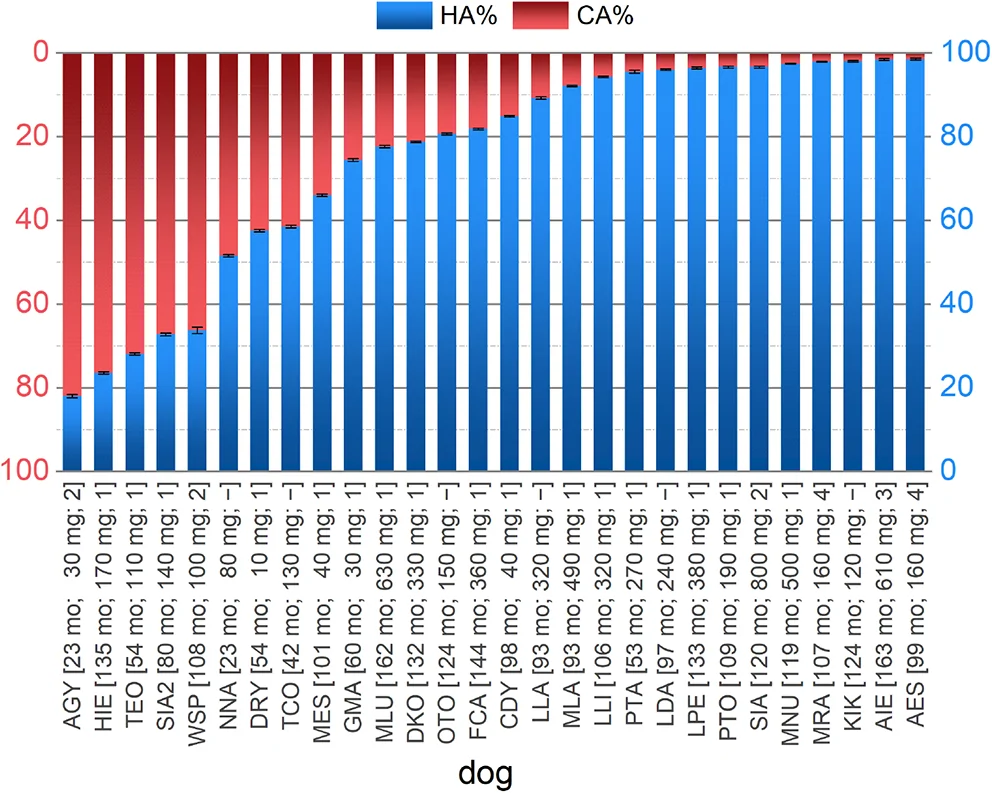
Variation of the HA% and CA% and estimated standard deviations within the crystalline fraction of the complete sample series. Dog age (months, mo), ablated calculus mass (mg), and oral-health score (0 = absence of pathologies; 1 = gingivitis; 2 = modest periodontitis; 3 = periodontitis; 4 = severe periodontitis; – = N/A) are indicated within square brackets (see Table S1).

Raman spectroscopy was used as a complementary phase-sensitive probe to support the identification of carbonate- and phosphate-bearing components and, through analysis of the phosphate ν_1_ band, to assess the structural order of the calcium-phosphate fraction. The results obtained for the LLI sample are reported in Figure 4, covering the spectral range from 200 to 2000 cm^–1^. The most intense peaks, clearly emerging from a broad fluorescence background, are assigned to phosphate (∼960 cm^–1^) and carbonate (∼1070 cm^–1^). Additionally, less intense features associated with the bending modes of phosphate around 400 and 600 cm^–1^ can be observed,^22,23^ along with two weaker bands (CH_2_ wagging and Amide I) attributed to proteins in hydroxyapatite of biogenic origin.^22^ Additionally, a weak peak at 1004 cm^–1^ can be tentatively attributed to phenylalanine.^24^

**Figure 4:**
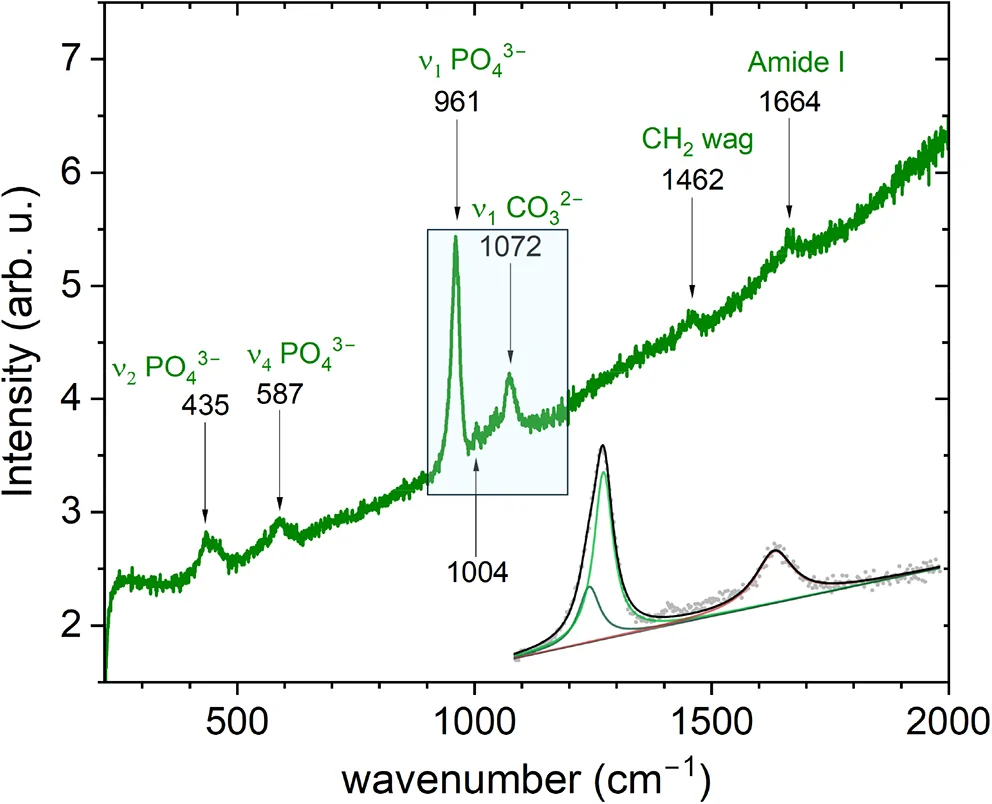
Raman spectrum of the LLI sample (405 nm excitation, ca. 10 mW power at the sample, 50× objective). Black arrows indicate the wavenumber position of the identified bands (black numbers) and chemical groups involved (green). The inset shows the Raman spectral fitting of the phosphate and carbonate ν_1_ region (900–1200 cm^–1^). The experimental data (gray dots) are described by three Lorentzian peaks at 950, 960 cm^–1^, and 1072 cm^–1^ (dark green, light green, and red), whose sum (black line) reproduces the observed profile with *R* ^2^ values exceeding 0.98.

On a closer look, the phosphate band appears structured and displays two main components, one at 950 cm^–1^, assigned to the amorphous fraction of the sample, and another at 960 cm^–1^, assigned to the crystalline part (Figure S4).^25^ Indeed, the HA crystalline phase often derives from a less ordered, metastable precursor known as amorphous calcium phosphate (ACP, Ca_*x*_(PO_4_)_*y*_·*n*H_2_O; *n* = 3–4.5). The relative amount of ACP and HA can be effectively monitored by Raman spectroscopy, since the phosphate symmetric stretching band shifts from approximately 950 cm^–1^ (amorphous phase) to 960 cm^–1^ (crystalline hydroxyapatite), accompanied by a progressive narrowing of the peak. This diagnostic behavior is described in ref 25 and provides a reliable spectroscopic approach to characterize the amorphous and crystalline phosphate fractions. For each spectrum, the region of the phosphate band (900–1020 cm^–1^) was selected, and the signal was deconvoluted into two Lorentzian components centered at ∼950 cm^–1^ and ∼960 cm^–1^, corresponding respectively to the amorphous (A) and crystalline (C) phosphate contributions. The linear baseline was subtracted, and fits were performed with *R* ^2^ > 0.98, following the procedure outlined in ref 25. The ratio of the areas of the amorphous and crystalline components (A/C = A_950_/A_960_) was then used as a semiquantitative indicator of the relative amorphous content in each specimen. The individual fits are shown in Figure S4 for the HA-rich samples LLI, LPE, SIA and MRA, and the results of the fitting for all spectra are summarized in Table S4. The distribution of the A/C ratios among the four samples is illustrated in Figure S5 as a bar chart. The data indicates some differences in phosphate ordering; for instance, the LLI sample exhibits a more disordered, less crystalline phosphate structure, as compared with the SIA and LPE samples. In the LLI sample, the Raman analysis allows us to conclude that, within the total amount of Ca-phosphate, 31% is constituted by ACP. Since the weight fraction of crystalline HA estimated in LLI is 37% (Table S2b), the weight fraction of ACP is deduced to be 17%. The remaining amorphous fraction – the overall amount estimated by Rietveld refinement is ∼61.4% – comes from other phases, probably of biogenic origin (*vide infra*). The ACP fractions in Ca-phosphate of samples LPE, SIA and MRA are 17%, 20% and 25%, respectively (Table S4).

As shown in the Figure S6, the carbonate band at ∼1070 cm^–1^ actually comprises two distinct contributions: one at ∼1072 cm^–1^ associated with carbonate ions substitutional to phosphate (B-type) within the hydroxyapatite lattice or to amorphous calcium carbonate (ACC),^23,26^ and another from carbonate ions associated with magnesian calcite (∼1086 cm^–1^).^27^ To investigate the possibility of qualitatively assessing the variations of the relative amounts of all carbonate and all phosphate groups by Raman, we collected spectra in the 900–1200 cm^–1^ region for the LLI, SIA, LPE, and MRA samples. We performed spectral fitting over a linear baseline to evaluate the area of the carbonate band (∼1070 cm^–1^) and compare it (carbonate/phosphate ratio) to that of the phosphate band (∼960 cm^–1^). The spectral fits are presented in Figure S7a, whereas the carbonate-to-phosphate band area ratio is shown in Figure S7b. We observe that the carbonate/phosphate ratio monotonically decreases from 0.45 to 0.20 along the sequence LLI, SIA, LPE, MRA (Figure S7b), whereas the CA/HA ratio obtained from XRD data is virtually constant (Figure 3). However, it is essential to emphasize the fundamental difference between the two ratios obtained using these two techniques: in XRD, the ratio represents the relative proportions of the CA and HA crystalline phases, whereas in Raman spectroscopy, it reflects the relative amounts of carbonate and phosphate anions.

### 3.2 Assessment of Correlations between Mineralogical and Elemental Composition of Dental Calculi – Monovariate Statistical Analysis

Some correlations can be expected to link the mineralogical composition (relative fraction of HA and CA) to the elemental composition. Assuming a composition of exclusively CA and HA, with Ca contributing to 40%_w/w_ both in CA and in HA, and with P contributing to 18.5%_w/w_ in HA, these relationships are expected:

$$\frac{\mathrm{Ca}}{P}=2.16\left(\frac{100}{\mathrm{HA}\%}\right);P\;\%=0.185\;\mathrm{HA}\%$$

The content of major elements and Mg of the ablated calculi is reported as weight percent as a function of apatite content in Figure 5. Calcium is the most abundant element with a fraction of 28% ± 9%, whereas the Mg content sets to 0.34% ± 0.12%. It is worth noting that Ca% and Mg% are not correlated with the apatite content, whereas P% augments monotonically but not linearly (Pearson’s *r* = 0; Spearman *ρ* = 0.536, *p* = 0.0122, Table 1) from 0.5% to 1.5% with the apatite content.

**Figure 5:**
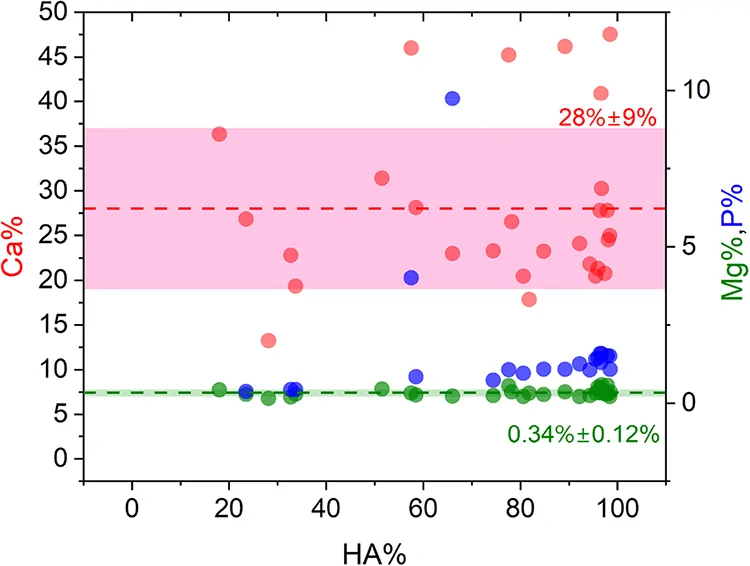
Weight percent of Ca (red dots, left axis), Mg (green dots, right axis), and P (blue dots, right axis) in the analyzed calculi as a function of their HA content. The dashed lines indicate the average value, and the shaded bands delimit the interval of ± one standard deviation. Experimental uncertainties are within the symbol size.

**Table 1:** Estimated Correlations between HA% and Elemental Composition of Canine Calculi

Relation	test	coefficient	*p*-value	comment
P% vs HA%	Spearman	*ρ* = 0.536	*p* = 0.0122	significant, monotone
Ca/P vs 1/HA%	Pearson	*r* = 0.713	*p* = 0.000285	Strong and significant

Figure 6a shows the fractions Ca/Mg and Ca/P as a function of the apatite content. While the ratio Ca/Mg is not correlated to HA% (it sets to 87 ± 26), the Ca/P ratio shows a decreasing trend with HA%, with a strong linear correlation with 1/HA% (Figure 6b; *r* = 0.713, *p* = 0.000285, Table 1). Considering that the Ca/P weight ratio and P% expected for pure HA are 2.16 and 18.5, respectively, while the corresponding estimated experimental values are 13 and 1.5, a significant portion of Ca must be hosted outside crystalline HA, including the crystalline carbonate phase identified by XRPD and potentially noncrystalline Ca-bearing components. On the light of the strong band at 1072 cm^–1^ observed in Raman spectra (Figures 4 and S6), this fraction of amorphous phase may include ACC.

**Figure 6:**
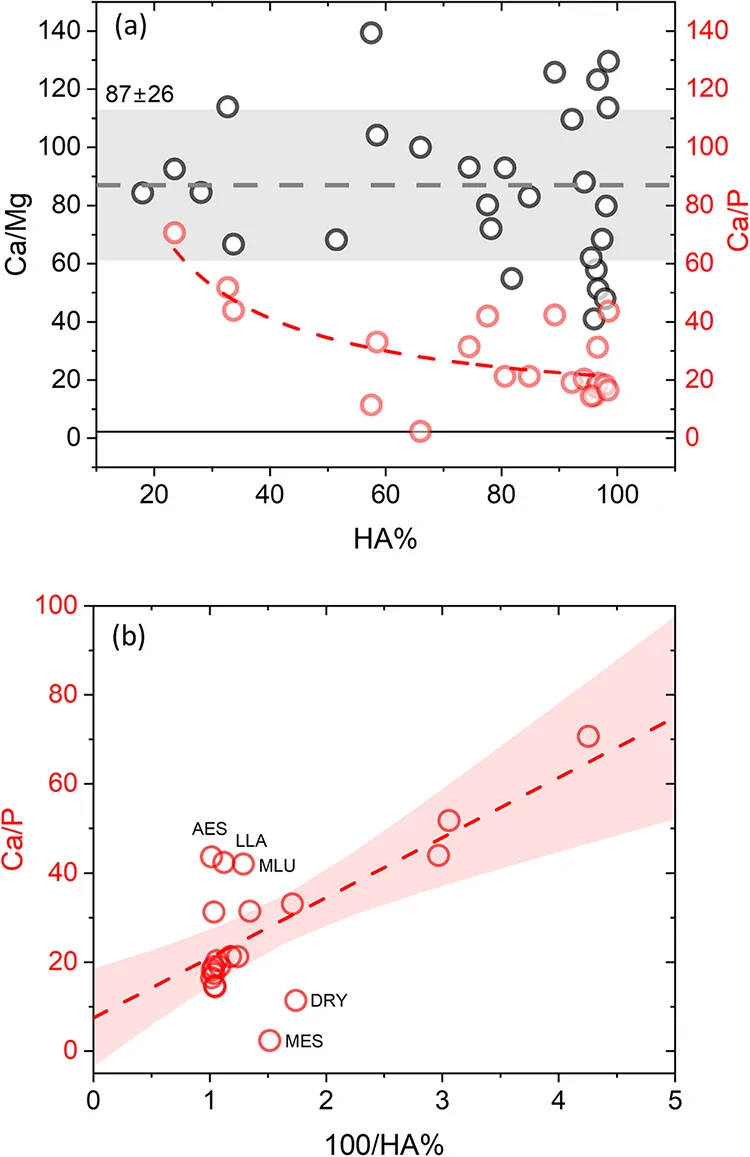
(a) Weight ratios of Ca/Mg (black circles) and Ca/P (red circles) in the analyzed calculi as a function of their HA content. The dashed horizontal gray line indicates the average value of Ca/Mg, and the shaded band delimits the interval of ± one standard deviation. The dashed red curve represents the best fit of the function Ca/P = *A* + *B*(1/HA%), with *A* and *B* fitting parameters. The solid horizontal line indicates the Ca/P ratio of pure HA (2.16). (b) Ca/P as a function of 100/HA% with linear regression (dashed red line, slope = 13 ± 3; Pearson’s *r* = 0.713, *p* = 0.000285). Experimental uncertainties are within the symbol size. The shaded pink area represents the 95% confidence band. The dog IDs of five outliers are indicated.

### 3.3 Assessment of Correlations between Calculus and Dog Specificities – Monovariate Statistical Analysis

The strongest correlation is observed between ablated mass and age/months from last ablation (Figure 7).

**Figure 7:**
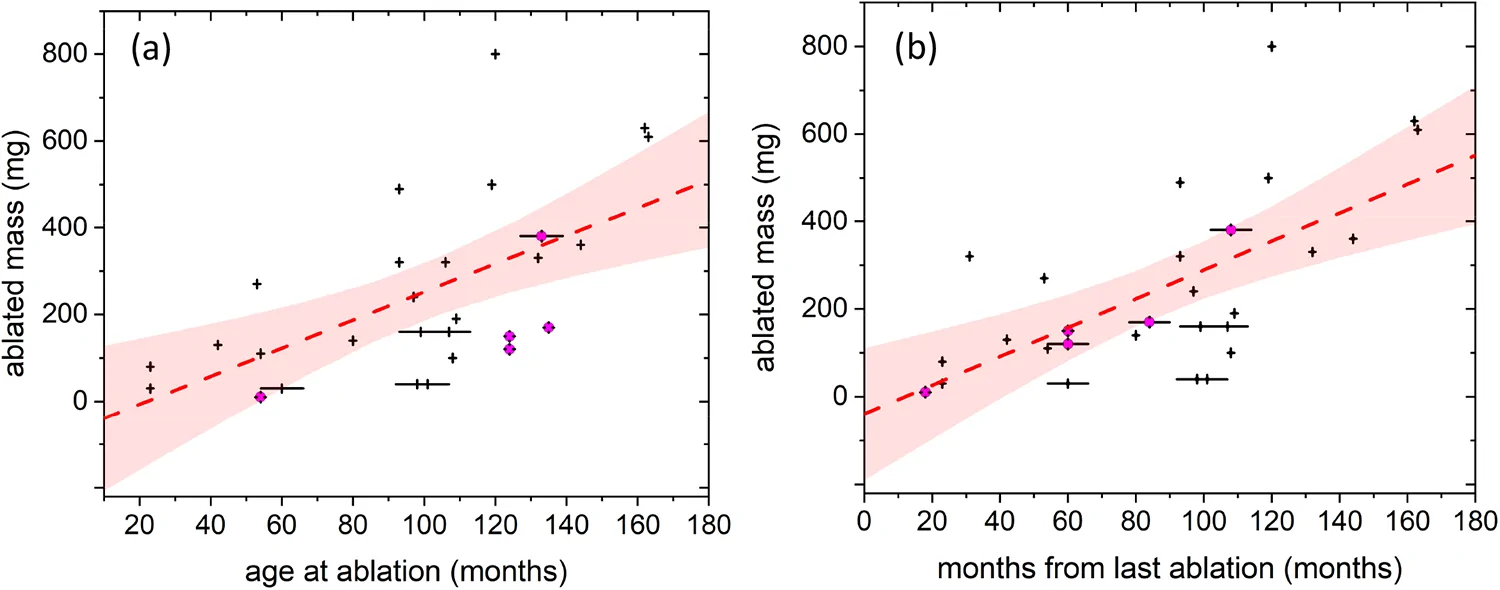
Ablated calculus mass as a function of the (a) dog age and (b) months from last ablation. The solid red lines represent linear regressions ((a): *r* = 0.599, *p* = 0.00076; *ρ* = 0.612, *p* = 0.00054; (b): *r* = 0.645, *p* = 0.00021; *ρ* = 0.652, *p* = 0.000172). Purple symbols identify dogs who were subjected to a previous ablation. The shaded pink areas represent the 95% confidence band.

The ablated calculus mass correlates more strongly with the time elapsed since the previous dental cleaning than with chronological age, indicating that the amount of collected calculi is associated with the estimated accumulation time of the deposit.

In relation to possible correlations between mineral composition and the specificity of the dogs, a significant but moderate correlation is also assessed between HA% and the ablated calculus mass (Table 2 and Figure S8), time elapsed from a previous ablation (if present, Figure S9) and dog age (Table 2 and Figure 8). Variable that does not show significant correlation with HA% (together with Ca% and Mg% and Ca/Mg mentioned in section 3.2) is dog weight.

**Figure 8:**
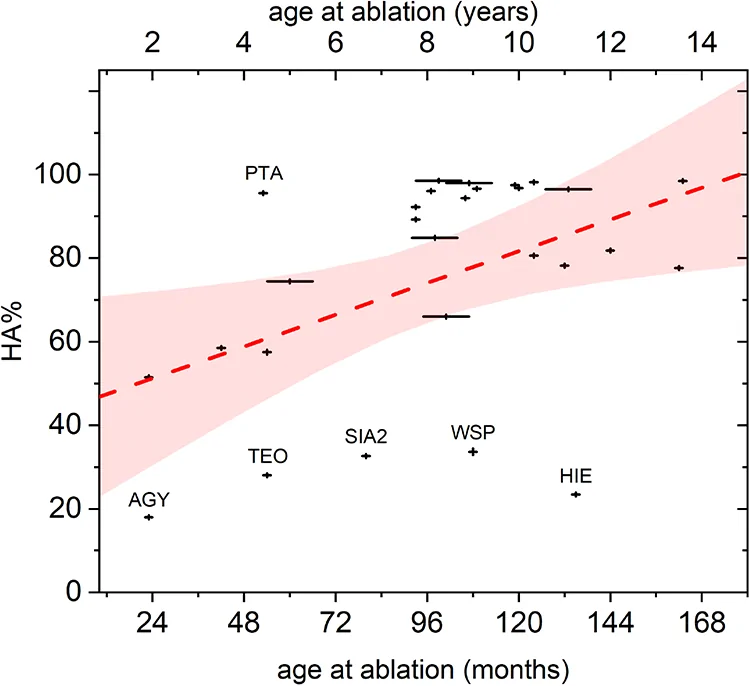
Weight percent of HA as a function of the dog age. The thick red dashed line represents the linear regression line of the data giving a HA% increase rate of (3.8 ± 1.4)%/year. The shaded pink area represents the 95% confidence band. The dog IDs of six outliers are indicated.

**Table 2:** Estimated Correlations between HA% and Some Dog Specificities

Relation	test	coefficient	*p*-value	comment
Ablated mass vs HA%	Pearson	*r* = 0.497	*p* = 0.0071	significant
	Spearman	ρ = 0.548	*p* = 0.0025	significant
Months from last ablation vs HA%	Pearson	*r* = 0.407	*p* = 0.0315	significant
	Spearman	ρ = 0.455	*p* = 0.0150	significant
Age vs HA%	Pearson	*r* = 0.455	*p* = 0.0149	significant
	Spearman	ρ = 0.403	*p* = 0.0333	significant

Principal outliers in respect to the HA%-age relationship are five dogs for their anomalously high CA% in their calculi (AGY, TEO, SIA2, WSP, and HIE), and one dog for its anomalously high HA% (PTA). The calcite-rich negative outliers are characterized by relatively low ablated calculus masses (Table 3) and by mild-to-moderate oral diseases (Figure 3).

**Table 3:** Distribution of Ablated Calculus Mass among the Considered 28 Dogs Divided in a Group of Calcitic Outliers (See Figure 7) and All Other Dogs

Group	*n*	Average mass (mg)	Median mass (mg)	Range (mg)
**Calcitic outliers**	5	110	110	30–170
**others**	23	277	240	10–800

To increase the group of positive outliers and balance the comparison, next to PTA we can include AES, MRA and SIA (Figure 3). These outliers are associated with relatively high ablated masses of 160, 160, and 800 mg, respectively, and their oral health is associated with severe periodontitis or mild periodontitis (Figure 3). Among the outliers of Figure 7, only AES appears as outlier in Figure 6b as well. This means that strong deviations of the HA content in relation to age are not associated with anomalous Ca/P ratios.

Since HA% also increases with ablated mass (Figure S8, Table 2), the apparent age dependence of the HA enrichment (Figure 8) should be interpreted as reflecting a combined effect of dog age, calculus accumulation history, and mineral maturation.

### 3.4 Assessment of Correlations between Calculus and Dog Specificities – Multivariate Statistical Analysis

Exploratory multivariate analysis further supported the existence of a coupled age–accumulation–composition trend. A multiple linear regression model using dog age, time elapsed since the previous dental cleaning and ablated calculus mass as predictors of the XRPD-derived crystalline HA fraction explained approximately 29% of the variance in HA% (*R* ^2^ = 0.287; adjusted *R* ^2^ = 0.198; model *p* = 0.0405). However, none of the individual predictors retained statistical significance when considered simultaneously (Table S5), indicating that age, accumulation time and ablated mass are partially interdependent descriptors rather than independent determinants of the mineral composition. Variance inflation factors remained moderate, supporting the use of the model as an exploratory description of covariation. When the ordinal oral-health score was added to the model in the subset of dogs with available oral information, the explained variance increased only slightly and the model was not statistically significant, confirming that the available clinical descriptors should be interpreted cautiously and not as formal periodontal staging.

Principal component analysis of the four core quantitative variables available for all dogs (age, time elapsed since the previous dental cleaning, ablated mass and HA%) identified a dominant first component explaining 68.4% of the total variance. This component showed positive loadings for all four variables, consistent with a common age–accumulation–mineral maturation axis (Table S6). A second component, explaining 17.0% of the variance, mainly separated samples with relatively high HA% from those with comparatively higher age or accumulation time but lower HA%. This second axis therefore highlights samples deviating from the main maturation trend, including calcite-rich samples with relatively low HA% for their age or accumulation history and apatite-rich samples with comparatively high HA% despite limited accumulation-related descriptors. A second PCA performed on the subset with complete chemical data showed that HA%, 100/HA%, Ca/P and P% contributed strongly to the compositional variance, consistent with major-element chemistry providing an independent descriptor of the calcite–apatite balance.

## 4 Discussion

The examination by XRPD and Raman spectroscopy of canine calculi (Figures 1–4) allows to establish that the mineral forms present therein are constituted by hydroxyapatite, Mg-calcite, and an amorphous phase. In the selected HA-rich specimens analyzed by Raman spectroscopy, the phosphate ν_1_ band indicates that part of the calcium phosphate component is structurally disordered and compatible with ACP-like contributions. While these findings corroborate HA as the dominant apatitic forms in dog DC,^10,14^ Mg-calcite was never mentioned as DC component. This represents an important increment of the knowledge of the composition of dog calculi, even though Mg occurs with concentrations almost 2 orders of magnitude lower than Ca (Figure 5). The predominance of hydroxyapatite among the crystalline calcium-phosphate phases detected in the present canine samples may reflect the analysis of relatively mature bulk deposits. It does not exclude the transient formation of other metastable calcium-phosphate phases during earlier stages of calculus mineralization.

Thanks to the consideration of a sufficiently large population of pet dogs, we demonstrated that HA and CA phases occur virtually in all proportions (Figures 2 and 3), contributing in solving the debate about the composition of canine calculi.^16^ Indeed, the contrasting results reported in the literature, with some claiming a calcitic composition and some claiming an apatitic composition, derives from the limited group of analyzed samples.

The relative amount of the calcitic and apatitic components depends on the specificity of the dog. To understand this link, consider that the strongest correlation is assessed between the mass of ablated calculi and the time elapsed from previous ablation treatments (Figure 8). The HA content, in turn, shows a clear correlation with the ablated mass (Table 2) and dog age (Table 2, Figure 7), while it is uncorrelated with Ca%, Mg%, Ca/Mg, dog sex, breed and weight. The linear regression of the age-HA% data allows to estimate the rate of HA increment as ∼4% y^–1^, with a lower limit of apatite content which probably hardly sets below 20%.

As the literature illustrates, pet dogs are naturally subjected to more severe PD stages as their age increases.^18^ In the light of our results, being PD closely related to DC, the progressive increment of calculus mass and dominance of the HA component with age goes in parallel with a worsening of the oral health. The motivations behind the increment of HA component can originate from the established negative correlation between serum Ca^2+^ levels (affecting in turn Ca levels in saliva) and dog age (independently of sex and weight).^17^ Hence, low Ca levels in saliva may favor the formation of HA. However, it is worth observing that Ca and Mg levels in DC (Figure 5), together with the Ca/Mg, Ca/P, and P%, display no significant correlation with dog age. Note that Ca/P is much higher than that expected for pure HA (2.16), indicating that a substantial fraction of Ca is hosted outside crystalline HA as noncrystalline Ca-bearing components of organic origin^14^ and possibly in the form of ACC, which is well recognized as a primary precursor phase in biomineralizations.^26^

The multivariate analysis clarifies the meaning and the limitations of the univariate correlations. The fact that HA enrichment correlates individually with age, time elapsed since the previous dental cleaning and ablated calculus mass, but that these variables do not behave as independent predictors in the multiple regression model, indicates that canine DC composition is better described by a coupled maturation/accumulation process than by a single controlling factor. In this framework, dog age likely captures biological and oral-environmental changes associated with aging, whereas ablated mass and time since previous cleaning describe the effective accumulation history of the deposit. PCA supports this interpretation by identifying a dominant axis along which age, accumulation descriptors and HA% covary (Tables S5 and S6). Samples deviating from this axis are particularly informative in defining the variability of the data set. Calcite-rich deposits in older dogs or after longer accumulation times, as well as apatite-rich deposits in younger dogs, may represent deviations from the general age-accumulation-composition relationship. However, because the study is cross-sectional and lacks standardized periodontal staging and calculus-free controls, these deviations should be regarded as hypotheses for future longitudinal studies rather than as diagnostic classifications.

The stronger correlation of apatite content with age than with time elapsed since the last ablation (Figure S9) suggests that DC composition does not simply reflect the chronological growth time of the deposit, but is also influenced by age-related biological and oral-environmental factors. Consistently, the relationship between the Ca/P ratio and the inverse apatite fraction (Figure 6) supports the use of major-element chemistry as an independent proxy for the calcite–apatite balance.

## 5 Conclusions

This study shows that canine DC is a chemically and mineralogically heterogeneous biomineral system rather than a deposit with a fixed crystalline composition. XRPD analysis identifies hydroxyapatite and magnesium-calcite as the main crystalline phases, occurring across a broad calcite–apatite continuum. Quantitative phase analysis of a representative sample and Raman spectroscopy of selected apatite-rich specimens further indicate that a substantial amorphous contribution is present, including structurally disordered phosphate components compatible with ACP and carbonate contributions consistent with ACC.

The combination of XRPD and ICP-OES demonstrates that the XRPD-derived crystalline hydroxyapatite fraction is reflected in the elemental composition of the deposits. In particular, phosphorus increases monotonically with hydroxyapatite enrichment, whereas calcium and magnesium remain comparatively constant. The markedly high Ca/P ratios, together with their nonlinear decrease with increasing hydroxyapatite fraction, indicate that a significant fraction of calcium is not hosted by crystalline hydroxyapatite, supporting the presence of calcium-bearing carbonate and amorphous components. Major-element chemistry therefore provides an independent descriptor of the calcite–apatite balance in canine DC.

Correlation and exploratory multivariate analyses indicate that hydroxyapatite enrichment is moderately associated with dog age, ablated calculus mass, and estimated accumulation time. These variables do not act as independent predictors; rather, they covary along a combined age-accumulation-composition axis. The observed shift from calcite-rich to apatite-rich deposits is consistent with a mineral-maturation process, but the cross-sectional design of the study does not establish a temporal calcite-to-apatite progression.

The mineralogical trends reported here should not be interpreted as a validated diagnostic or prognostic tool. Rather, they establish a mineralogical and chemical framework for canine dental calculus and provide a basis for future controlled studies aimed at resolving site-specific heterogeneity, temporal mineral maturation, and the possible relationship between calculus composition and canine oral health. Notwithstanding these limitations, deviations from the expected mineralogical maturation trend, such as advanced apatite-rich mineralization in young dogs or disproportionate calculus accumulation over short time intervals, provide testable hypotheses for future longitudinal studies aimed at determining whether departures from the age-accumulation-composition relationship are associated with differences in mineral maturation or oral-disease progression.

## Supplementary Material



## Notes

Information collected for the 28 analyzed dogs (Table S1); quantitative XRPD phase analysis (Figure S1, Tables S2a, S2b and S3); XRPD of the whole set of samples (Figure S2). Distribution of extracted HA% (Figure S3); raman spectral fitting in the phosphate region, estimation of the amorphous/crystalline and carbonate/phosphate ratios (Figures S4–S7, Table S4); linear fit of ablated calculus mass vs HA% (Figure S8); linear fit of HA% vs months from previous dental cleaning (Figure S9); summary of the results of the exploratory multivariate statistical analysis (multiple linear regression models and principal component analysis) (Tables S5,S6) (PDF)
