# Supplementary material for: Mineralogical and Elemental Variability of Canine Dental Calculi Reveals an Age-Associated Calcite–Apatite Continuum
Source: ACS Omega. 2026 Oct 9;11(41):63412–22. doi: 10.1021/acsomega.6c07170 (PMC13689202; doi:10.1021/acsomega.6c07170)
Supplement: Supplementary file 1 [file ao6c07170_si_001.pdf]

Supporting Information

Mineralogical and Elemental Variability of Canine Dental Calculi Reveals an Age-Associated Calcite–Apatite Continuum

Marcello Campione[1,*], Andrea Lucotti[2], Matteo Tommasini[2], Claudio Pezzoli[3], Valerio Cerantola[1], and Giancarlo Capitani[1]

[1]Department of Earth and Environmental Sciences, University of Milano - Bicocca, Italy.

[2]Department of Chemistry, Materials, and Chemical Engineering, Politecnico di Milano, Italy.

[3]Clinica Veterinaria Bicocca, Viale Fulvio Testi, 177, 20162 Milano, Italy

*Corresponding Author. Email: marcello.campione@unimib.it

Table S1. Specific pieces of information collected for the analyzed dogs subjected to dental calculus ablation. Samples are ordered with increasing HA%, as determined by XRPD-QPA (Figure S1). Shaded cells indicate estimated age within ±6 months. Oral-health score: 0 = absence of pathologies; 1 = gingivitis; 2 = modest periodontitis; 3 = periodontitis; 4 = severe periodontitis.

Sample ID	Ablation Date	Date of Birth	Age at Ablation (months)	Months from Previous Ablation	Breed	Weight (kg)	Sex / Reproductive Status	Ablated mass (mg)	HA%	Diet Type	Pathologies (systemic/oral [health score])	Main Locations Frequented
240913 AGY	13-06-24	30-06-22	23	23	Belgian Shepherd	21.2	Female	30	18.0 ± 0.4	Commercial food (dry + wet)	Modest periodontitis [2]	Milan city
240913 HIE	13-09-24	15-05-13	135	84	Jack Russel Terrier	8.7	Female	170	23.5 ± 0.3	Commercial food (dry + wet)	Atopic Dermatitis/ Gingivitis [1]	Milan city
240522 TEO	22-05-24	20-11-19	54	54	Mixed breed	13.6	Male, castrated	110	28.1 ± 0.3	Commercial food (dry + wet)	Gingivitis [1]	Milan city
250214 SIA2	12-02-25	15-05-18	80	80	Mixed breed	13.5	Female	140	32.7 ± 0.3	Commercial food (dry + wet)	Gingivitis [1]	Milan city
250122 WSP	02-01-25	26-12-15	108	108	Jack Russel Terrier	9.5	Male, castrated	100	33.7 ± 0.8	Prepared + commercial food	Modest periodontitis [2]	Milan city
240510 NNA	10-05-24	10-06-22	23	23	Maltese	N/A	Female	80	51.5 ± 0.3	Commercial food (dry + wet)	None, N/A	Milan city
240910 DRY	10-09-24	22-02-20	54	18	King Charles Spaniel	10.3	Female	10	57.5 ± 0.6	Commercial food (dry + wet)	Gingivitis [1]	Milan city
240905 TCO	05-09-24	19-02-21	42	42	Giant Poodle	N/A	Male	130	58.5 ± 0.3	Commercial food (dry + wet)	None/N/A	Milan city
240614 MES	16-06-24	01-01-16	101	101	Mixed breed	18.5	Male	40	66.0 ± 0.3	Prepared food	Gingivitis [1]	Milan city
250508 GMA	08-05-25	08-05-20	60	60	Mixed breed	7.3	Female	30	74.4 ± 0.3	Commercial food (dry + wet)	Gingivitis [1]	Milan city
260415 MLU	15-04-26	05-10-12	162	162	Mixed breed	10	Female	630	77.6 ± 0.3	Commercial food (dry + wet)	Moderate Gingivitis on upper right and left molars; absence of caries [1]	Milan city
240607 DKO	07-06-24	08-05-13	132	132	American Pit Bull	32.5	Male	330	78.2 ± 0.2	Commercial food (dry + wet)	Gingivitis [1]	Milan city
240717 OTO	17-07-24	28-02-14	124	60	Mixed breed	N/A	Male, castrated	150	80.6 ± 0.2	Commercial food (dry + wet)	N/A	Milan city
240520 FCA	20-05-24	04-05-12	144	144	Pug	7.8	Female, spayed	360	81.8 ± 0.2	Commercial food (dry + wet)	Gingivitis [1]	Milan city
250318 CDY	18-03-25	01-01-17	98	98	Mixed breed	16.0	Female	40	84.8 ± 0.2	Commercial food (dry + wet)	Gingivitis [1]	Milan city
260408 LLA	08-04-26	25-06-18	93	93	Mixed breed	8.0	Female, spayed	320	89.2 ± 0.3	Commercial food (dry + wet)	None/none	Milan city

241009 MLA	09-10-24	13-12-16	93	93	Maltese	3.8	Female, spayed	490	92.2 ± 0.2	Commercial food (dry + wet)	Gingivitis [1]	Milan city
241016 LLI	16-10-24	22-11-15	106	31	Poodle	7.6	Female	320	94.3 ± 0.2	Prepared + commercial food	Gastritis/ Gingivitis [1]	Milan city
241106 PTA	06-11-24	20-05-20	53	53	Mixed breed	11.3	Female, spayed	270	95.5 ± 0.4	Prepared + commercial food	Gingivitis [1]	Milan city
250218 LDA	18-02-25	16-01-17	97	97	Poodle	N/A	Female	240	96.0 ± 0.2	Commercial food (dry + wet)	None, N/A	Milan city
250218 LPE	18-02-25	01-01-14	133	108	Poodle	12.3	Male, castrated	380	96.4 ± 0.2	Commercial food (dry + wet)	Gingivitis [1]	Milan city
260416 PTO	16-04-26	03-03-17	109	109	Hound	18.0	Male	190	96.6 ± 0.2	Commercial food (dry + wet)	Moderate Gingivitis on upper right and left molars; presence of caries 2nd upper left molar [1]	Milan city
241105 SIA	05-11-24	01-11-14	120	120	Mixed breed	N/A	Female	800	96.7 ± 0.2	Commercial food (dry + wet)	Periodontitis [2]	Milan city
240508 MNU	08-05-24	22-05-14	119	119	Beagle	15.3	Female, spayed	500	97.4 ± 0.1	Prepared food	Gastritis/Gingivitis [1]	Milan city
241202 MRA	02-12-24	01-01-16	107	107	Schnauzer	10.5	Female, spayed	160	97.9 ± 0.1	Commercial food (dry + wet)	Severe Periodontitis [4]	Milan city
240606 KIK	06-06-24	16-01-14	124	60	Maltese	N/A	Male	120	98.1 ± 0.2	Prepared food	None, N/A	Milan city
241010 AIE	10-10-24	02-03-11	163	163	Mixed breed	14.4	Female, spayed	610	98.4 ± 0.2	Commercial food (dry + wet)	Gingivitis + modest periodontitis [3]	Milan city + countryside
260409 AES	09-04-26	01-01-18	99	99	Mixed breed	8.0	Male	160	98.5 ± 0.2	Commercial food (dry + wet)	Severe Periodontitis; absence of caries [4]	Milan city

XRPD Quantitative Phase Analysis (QPA)

The identification of hydroxiapatite and magnesian calcite (Mg-calcite) was based on the highest matching score provided by the X'Pert HighScore software (PANalytical), which makes use of the PDF database. In 22 DC samples out of 28 (79%), the best matching pattern for the crystalline phosphate was the hydroxiapatite by Hanawalt et al.,[1] ref. code 00-001-1008. In all the other cases, chlorapatite (3 times), carbonated hydroxyapatite (2), and fluorapatite (1) recorded the highest scores. In 23 DC samples out of 28, a crystalline carbonate was automatically detected by the software (those missed were almost pure in hydroxiapatite); among these 23 samples, 20 were Mg-calcite (87%) and only 3 calcite. The 20 spectra matching Mg-calcite were equally divided between the reference pattern by Paquette et al.,[2] (ICSD collection code 040108), and that by Falini et al.,[3] (ICSD collection code 086161). The reported chemical compositions were: $Mg_{0.064}Ca_{0.936}CO_3$ and $Mg_{0.042}Ca_{0.958}CO_3$, respectively. The reported cell parameters were: a = 4.967, c = 16.963; and a = 4.978, c = 16.988 Å, respectively. The average refined cell parameters for our DC are: a = 4.9845 and c = 17.0855 Å. The estimated $MgCO_3$ of calcite according to Fahad and Saeed[4] – using the

regression equation with strongest correlation, i.e. Mg Mol% vs. *a* – varies between 0.86 and 2.33 mol%, with an average of 1.16 mol%. On this basis, the mineral can be defined a low-Mg calcite.[5]

The quantitative phase analysis (QPA) was performed with the Rietveld method[6] using the Profex v. 5.6.1 software.[7] The initial structures of hydroxiapatite and Mg-calcite were selected from the Profex's internal structure database (ref. codes: amcsd_0001257 and 04-008-0788, respectively). To determine the amorphous content, ~10 wt.% of α-alumina (corundum) was added as an internal standard (ref. code: 04-004-2852). The background was modelled using a 8-term Chebyshev function. The *a* and *b* cell parameters, crystallite size and micro strain broadening effects were allowed to vary during refinement for all involved phases.

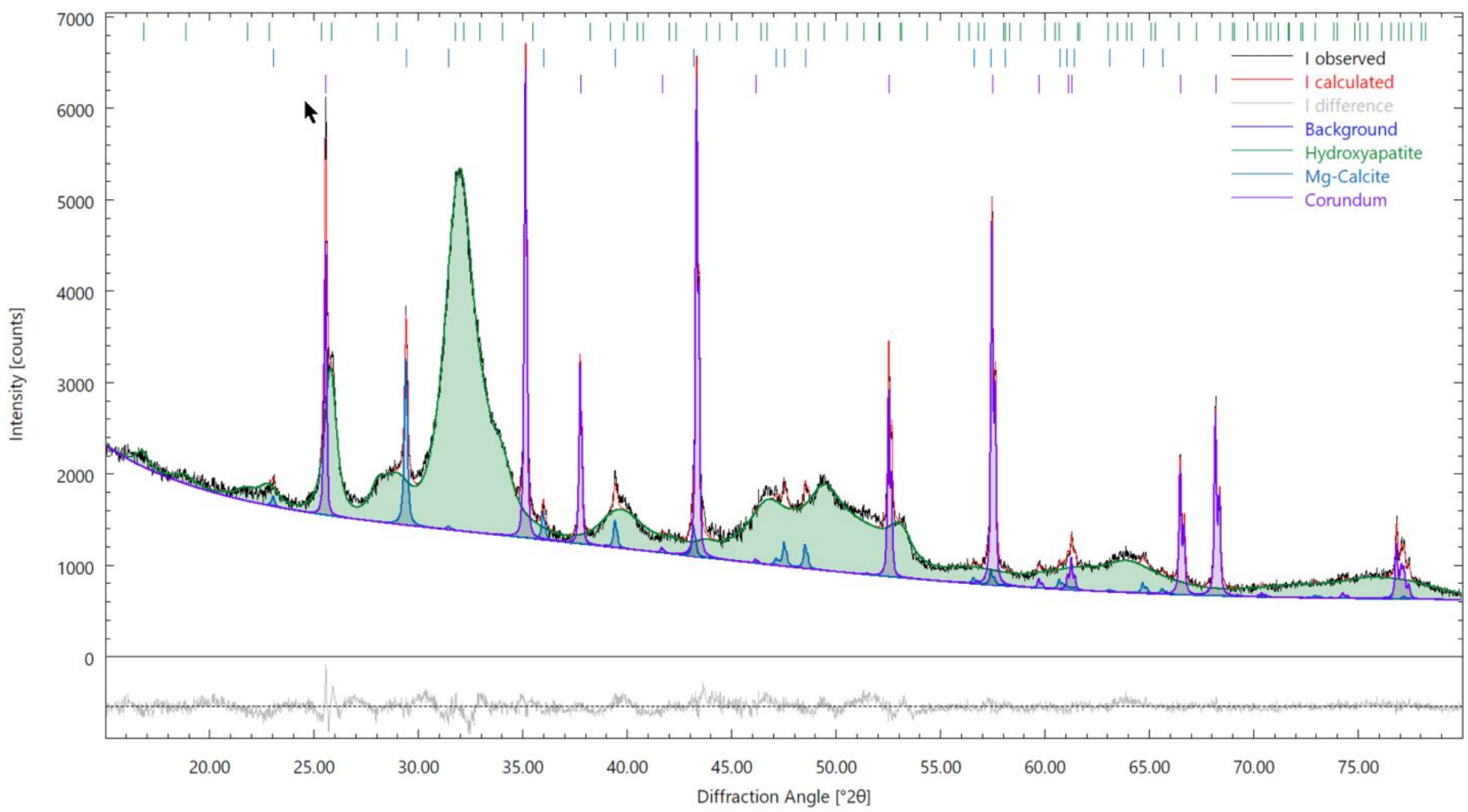

Figure S1. Experimental vs. calculated XRPD plot for the LLI sample with corundum as internal standard, along with calculated background and residues (lower rows).

Table S2a. *hkl* indexes, peaks positions and relative intensities for the XRPD pattern of the LLI sample with corundum (CO) as internal standard.

Phase	*h*	*k*	*l*	Angle (°2θ)	Rel. Int. (%)	Phase	*h*	*k*	*l*	Angle (°2θ)	Rel. Int. (%)
HA	1	-1	1	16.8298	3.03	HA	6	-3	0	58.8287	1.01
HA	2	-1	0	18.8443	2.52	CO	3	-2	-1	59.7396	0.50
HA	2	-2	0	21.7927	3.62	HA	6	-2	0	60.0177	4.25
HA	2	-1	1	22.8631	5.54	HA	6	-3	1	60.4861	4.10
CA	1	-1	2	23.0728	0.54	HA	4	-4	3	60.6953	0.02
HA	2	-2	1	25.3688	1.74	CA	3	-2	-4	60.7224	0.64
CO	1	-1	2	25.5761	10.69	CA	2	-2	-8	61.0481	0.26
HA	0	0	2	25.8231	28.19	CO	3	-2	2	61.1291	0.72

HA	1	-1	2	28.0818	5.89
HA	3	-2	0	28.963	16.69
CA	1	-1	-4	29.4227	9.11
CA	0	0	6	31.4567	0.20
HA	3	-2	1	31.7917	100.00
HA	2	-1	2	32.1624	36.95
HA	3	-3	0	32.9443	37.63
HA	2	-2	2	34.037	16.31
CO	1	-1	-4	35.1508	19.73
HA	3	-3	1	35.4882	2.79
CA	2	-1	0	36.0006	1.56
CO	2	-1	0	37.7759	7.78
HA	4	-2	0	38.2239	0.30
HA	3	-2	2	39.1881	4.35
CA	2	-1	3	39.4419	1.59
HA	4	-1	0	39.8493	16.15
HA	4	-2	1	40.4798	1.91
HA	1	-1	3	40.7597	0.22
CO	0	0	6	41.6791	0.22
HA	4	-1	1	42.0322	4.88
HA	3	-3	2	42.3246	0.90
CA	2	-2	-2	43.1958	1.85
CO	2	-1	3	43.3535	21.50
HA	2	-1	3	43.8032	3.61
HA	4	-4	0	44.4279	0.61
HA	2	-2	3	45.2629	2.72
CO	2	-2	-2	46.1776	0.20
HA	4	-4	1	46.4357	0.14
HA	4	-2	2	46.706	29.32
CA	2	-2	4	47.154	0.39
CA	1	-1	8	47.5408	1.45
HA	4	-1	2	48.0967	11.09
CA	2	-1	6	48.5439	1.50
HA	5	-2	0	48.6595	4.30
HA	3	-2	3	49.4392	24.50
HA	5	-3	1	50.5379	18.86
HA	5	-4	0	51.3326	8.80
HA	3	-3	3	52.0829	0.94
HA	4	-4	2	52.1007	9.38
CO	2	-2	4	52.5512	9.25
HA	0	0	4	53.0892	12.13
HA	5	-4	1	53.142	0.52
HA	1	-1	4	54.359	0.80
HA	5	-3	2	55.8975	6.81
CO	1	-1	8	61.3049	1.37
CA	2	-1	9	61.4231	0.20
HA	3	-1	4	61.6078	3.29
HA	6	-2	1	61.6558	2.80
HA	5	-5	2	63.0332	6.53
CA	3	-2	5	63.1087	0.14
HA	6	-5	0	63.5042	1.07
HA	3	-3	4	63.9107	5.47
HA	5	-3	3	64.1604	5.51
CA	3	-3	0	64.7206	0.75
HA	6	-5	1	65.0917	6.14
HA	6	-3	2	65.3079	1.56
CA	0	0	12	65.6608	0.38
HA	5	-1	3	66.4143	1.14
HA	6	-4	2	66.4297	1.32
CO	3	-2	-4	66.5178	7.23
HA	4	-2	4	67.2836	0.01
CO	3	-3	0	68.2081	10.85
HA	4	-3	4	68.3889	0.32
HA	1	-1	5	69.021	0.06
HA	6	-6	0	69.0963	0.17
CA	3	-2	-7	69.2481	0.12
HA	6	-5	2	69.7404	1.85
HA	7	-4	0	70.1882	0.42
CA	2	-2	10	70.2991	0.14
CO	3	-2	5	70.4162	0.25
HA	6	-6	1	70.6199	0.02
HA	5	-5	3	70.8129	0.19
HA	2	-1	5	71.1984	0.23
HA	4	-4	4	71.6571	0.23
HA	7	-4	1	71.7015	4.56
HA	2	-2	5	72.2764	0.18
HA	7	-5	0	72.3502	3.26
CA	3	-2	8	72.9571	0.20
HA	6	-3	3	72.9671	0.17
CA	3	-3	6	73.7358	0.07
HA	7	-5	1	73.8452	2.01
HA	6	-2	3	74.0348	3.47
CO	2	-2	-8	74.3024	0.35
HA	5	-2	4	74.8644	0.98
HA	6	-6	2	75.1114	0.82
HA	3	-2	5	75.4736	3.55
HA	7	-3	2	76.1681	2.91
CA	4	-2	0	76.3468	0.14

HA	5	-5	0	56.4041	0.05		HA	7	-6	0	76.601	1.29
CA	3	-2	-1	56.614	0.38		CO	1	-1	-10	76.8778	3.62
HA	2	-1	4	56.8405	0.10		HA	5	-1	4	76.9753	4.25
HA	4	-3	3	57.1082	3.34		HA	6	-5	3	77.2056	3.36
CA	3	-2	2	57.4504	0.93		CA	2	-1	12	77.2235	0.19
CO	2	-1	6	57.5004	18.95		CO	2	-1	9	77.2384	1.74
HA	2	-2	4	58.055	0.13		HA	3	-3	5	77.579	0.10
HA	5	-5	1	58.1048	1.14		HA	7	-1	1	78.0671	0.53
CA	1	-1	-10	58.1229	0.09		HA	7	-2	2	78.2676	5.63
HA	5	-4	2	58.3357	0.69							

Table S2b. QPA results and refinement parameters for LLI. Values are expressed as weight fractions (ESD: estimated standard deviations).

	LLI		LLI (with corundum)	
Phase/Parameter	Value	ESD	Value	ESD
Mg-Calcite	0.0468	0.0012	0.01759	0.00055
Hydroxyapatite	0.9532	0.0012	0.3684	0.0031
Amorphous	-	-	0.614	0.0033
R_{wp}	3.61		3.32	
R_{exp}	2.53		2.53	
χ^2	2.036		1.722	
GOF	1.4269		1.3123	

Table S3. QPA results and refinement parameters for all samples (estimated standard deviations in brackets). The parameters *a* refers to Mg-calcite and has been used to estimate $MgCO_3$ mol% (Mg mol%).

	**AGY**	**HIE**	**TEO**	**SIA2**	**WSP**	**NNA**	**DRY**
CA (wt.)	0.820(4)	0.765(3)	0.719(3)	0.673(3)	0.663(8)	0.485(3)	0.425(6)
HA (wt.)	0.180(4)	0.235(3)	0.281(3)	0.327(3)	0.337(8)	0.515(3)	0.575(6)
a (Å)	4.9893(1)	4.9865(1)	4.9889(1)	4.9897(1)	4.9873(1)	4.9893(1)	4.9893(2)
Mg mol%	1.02	1.10	1.03	1.01	1.08	1.02	1.02
R_{wp}	5.39	5.64	5.31	5.74	5.55	5.23	5.02
R_{exp}	4.53	4.72	4.55	4.69	4.75	4.44	4.43
Chi^2	1.42	1.43	1.36	1.50	1.37	1.39	1.28
GOF	1.19	1.19	1.17	1.22	1.17	1.18	1.13
	**TCO**	**MES**	**GMA**	**MLU**	**DKO**	**OTO**	**FCA**
CA (wt.)	0.415(3)	0.340(3)	0.256(3)	0.224(3)	0.218(2)	0.194(2)	0.182(2)
HA (wt.)	0.585(3)	0.660(3)	0.744(3)	0.776(3)	0.782(2)	0.806(2)	0.818(2)
a (Å)	4.9904(1)	4.9897(2)	4.9894(2)	4.9873(3)	4.9892(2)	4.9888(2)	4.9878(3)
Mg mol%	0.99	1.01	1.02	1.08	1.03	1.04	1.07
R_{wp}	5.28	5.06	5.09	5.19	4.99	4.97	5.21
R_{exp}	4.56	4.49	4.50	4.43	4.26	4.25	4.63
Chi^2	1.34	1.27	1.28	1.37	1.37	1.37	1.27
GOF	1.16	1.13	1.13	1.17	1.17	1.17	1.13
	**CDY**	**LLA**	**MLA**	**LLI**	**PTA**	**LDA**	**LPE**
CA (wt.)	0.152(2)	0.108(3)	0.079(2)	0.057(2)	0.045(4)	0.040(2)	0.036(2)
HA (wt.)	0.848(2)	0.892(3)	0.922(2)	0.943(2)	0.955(4)	0.960(2)	0.964(2)
a (Å)	4.9877(3)	4.9950(6)	4.9890(5)	4.9885(10)	4.9440(4)	4.9878(10)	4.9900(13)

Mg mol%	1.07	0.86	1.03	1.05	2.33	1.07	1.00
R_{wp}	5.03	5.05	5.21	5.02	4.89	5.39	5.31
R_{exp}	4.35	4.50	4.43	4.27	4.29	4.43	4.65
Chi^2	1.34	1.26	1.38	1.38	1.30	1.48	1.30
GOF	1.16	1.12	1.18	1.18	1.14	1.22	1.14

	**PTO**	**SIA**	**MNU**	**MRA**	**KIK**	**AIE**	**AES**
CA (wt.)	0.034(2)	0.034(2)	0.026(1)	0.021(1)	0.020(2)	0.016(2)	0.015(2)
HA (wt.)	0.966(2)	0.967(2)	0.974(1)	0.979(1)	0.981(2)	0.984(2)	0.985(2)
a (Å)	4.9887(30)	4.9894(9)	4.9903(8)	4.9891(20)	4.9644(69)	4.9590(77)	4.9608(103)
Mg mol%	1.04	1.02	0.99	1.03	1.74	1.90	1.85
R_{wp}	5.21	4.86	4.99	4.85	5.15	5.05	5.02
R_{exp}	4.56	4.24	4.18	4.27	4.54	4.41	4.35
Chi^2	1.31	1.31	1.43	1.29	1.29	1.31	1.33
GOF	1.14	1.15	1.19	1.14	1.13	1.15	1.15

Mg mol% = $-28.44009 * a + 145.41384$ (Fahad & Saeed, 2018).

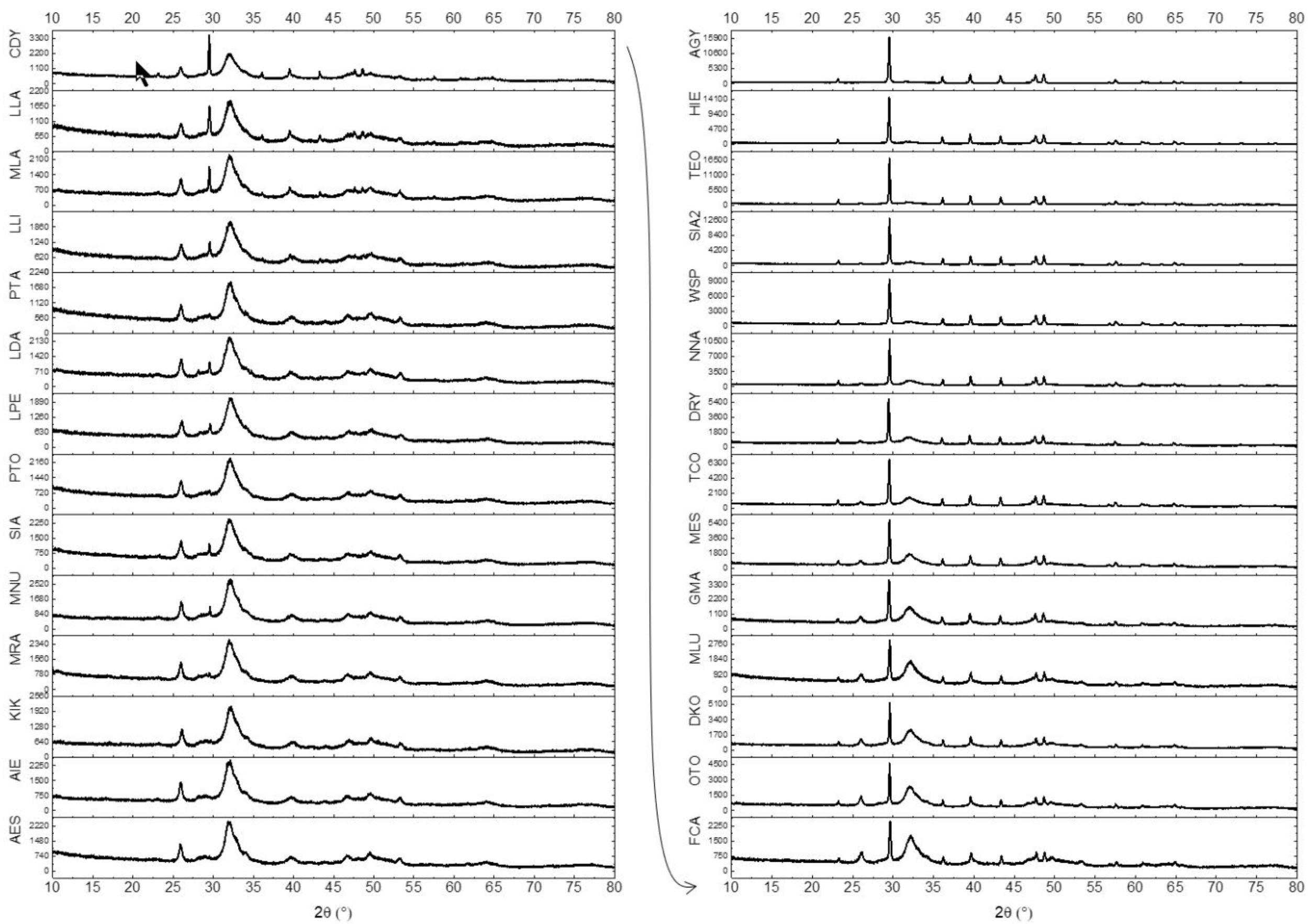

Figure S2. XRPD patterns of all 28 DC samples collected showing a continuous variation from almost pure HA composition (lower-left) through almost pure CA composition (upper-right), with few exceptions, i.e. PTA and PTO, which probably should occupy lower positions.

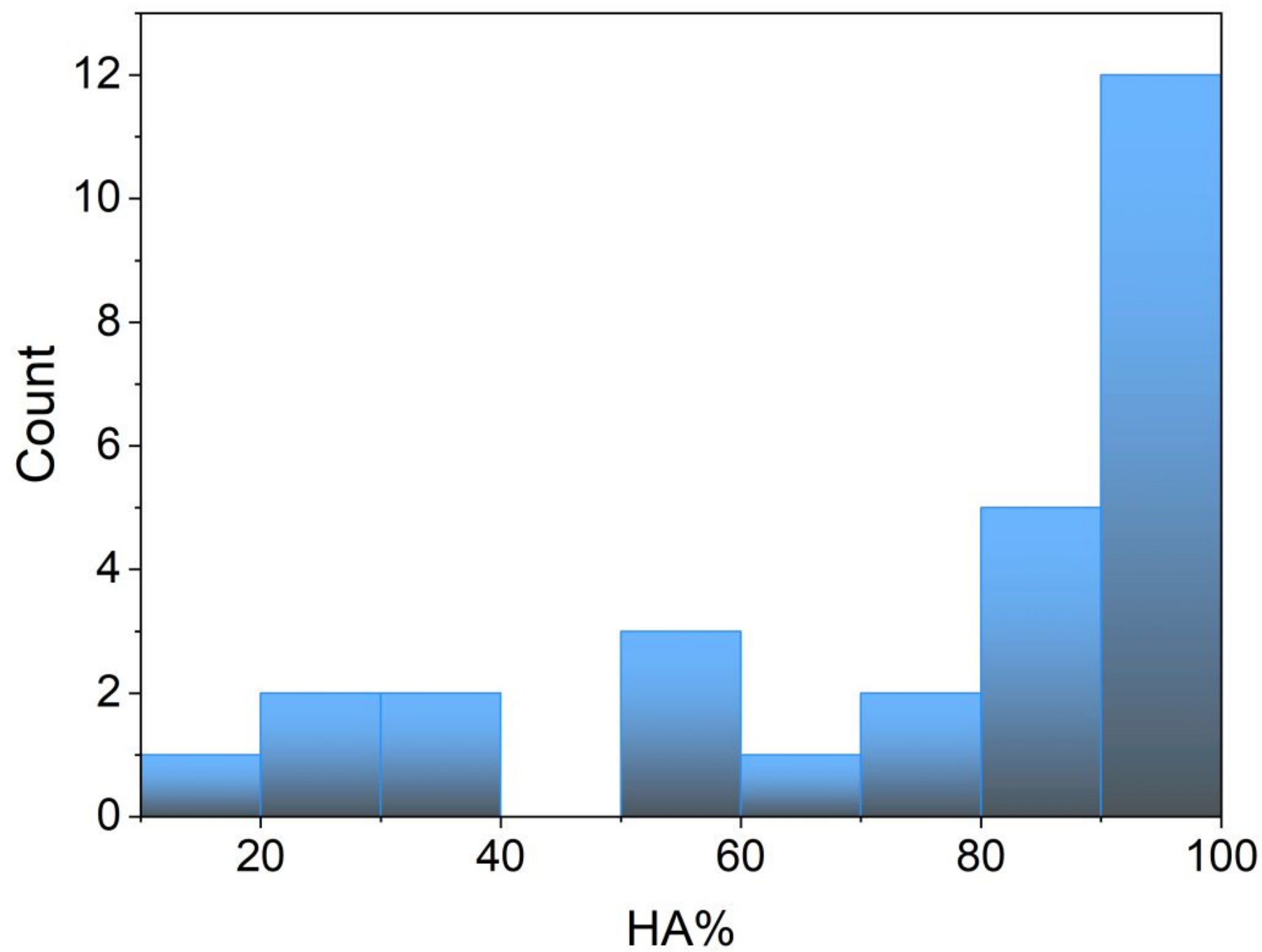

Figure S3. Distribution of HA% measured by XRPD-QPA within the group of 28 pet dogs (derived for Figure 3).

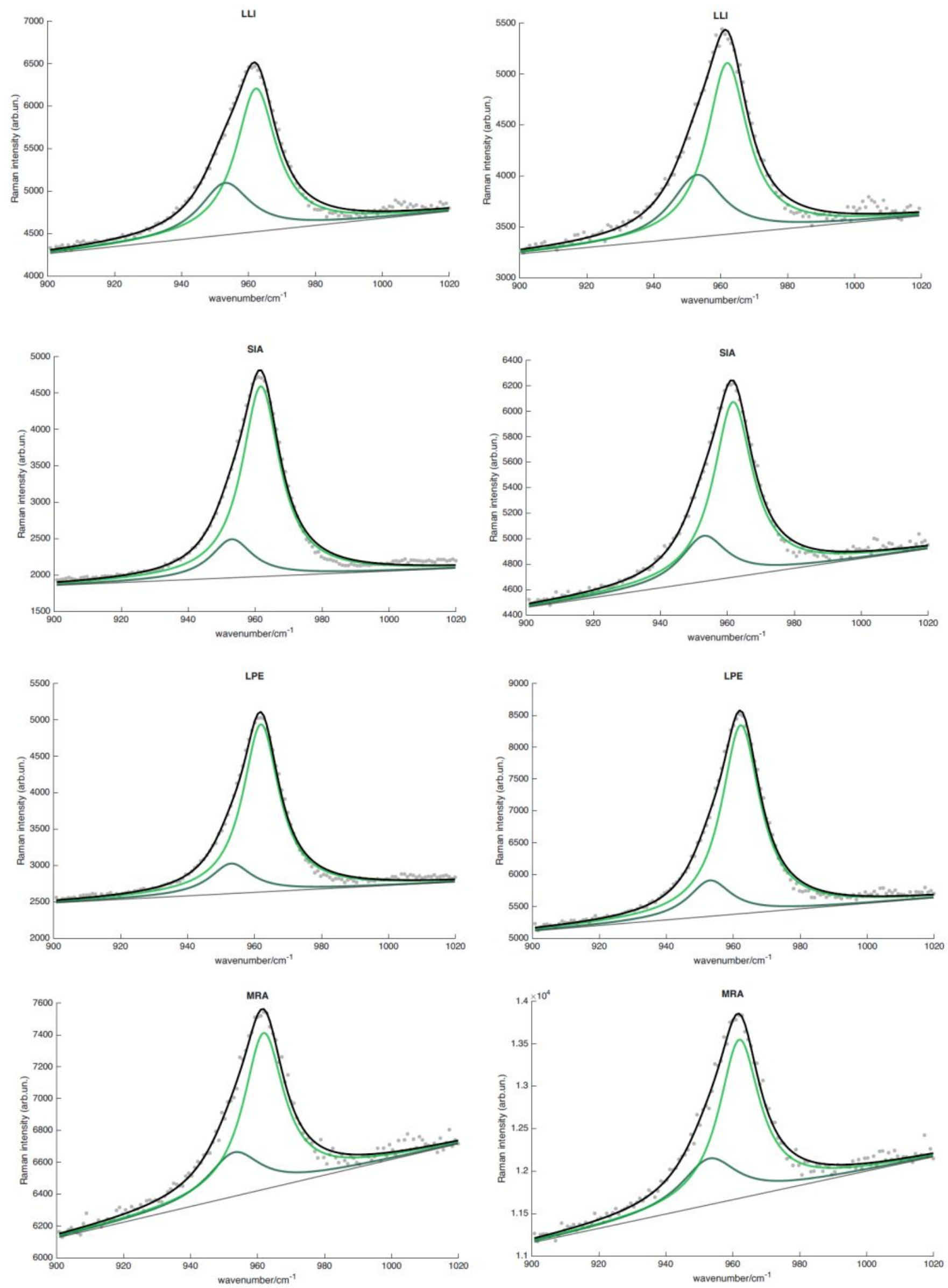

Figure S4. Example of Raman spectral fitting (two regions for each sample) in the phosphate ν_1 region (900–1020 cm^{-1}). The experimental data (grey dots) are described by two Lorentzian peaks at 950 and 960 cm^{-1} (dark and light green), whose sum (black line) reproduces the observed profile with R^2 values exceeding 0.98.

Table S4. Parameters obtained from the Lorentzian fitting of the phosphate band. The table lists for each sample the positions of the two components of the phosphate band (ν_1, ν_2), the full width at half maximum (γ_1, γ_2), and the resulting amorphous (A) / crystalline (C) ratio (A/C = A_{950}/A_{960}) and percentage of A phase (A%). The coefficient of determination (R^2) of the fitting is also reported. The mean A/C values were used for comparison among the samples.

sample	ν_1 (A)	ν_2 (C)	γ_1	γ_2	A/C	R^2	<A/C>	<A%>
**LLI**	953	962	18	15	0.39	0.98	0.44	31
**LLI**	953	962	20	15	0.49	0.98		
**SIA**	953	962	15	14	0.21	0.99	0.25	20
**SIA**	953	961	15	14	0.30	0.99		
**LPE**	953	962	15	14	0.17	0.99	0.20	17
**LPE**	953	962	16	14	0.23	0.99		
**MRA**	953	962	18	15	0.39	0.98	0.34	25
**MRA**	953	961	18	15	0.30	0.98		

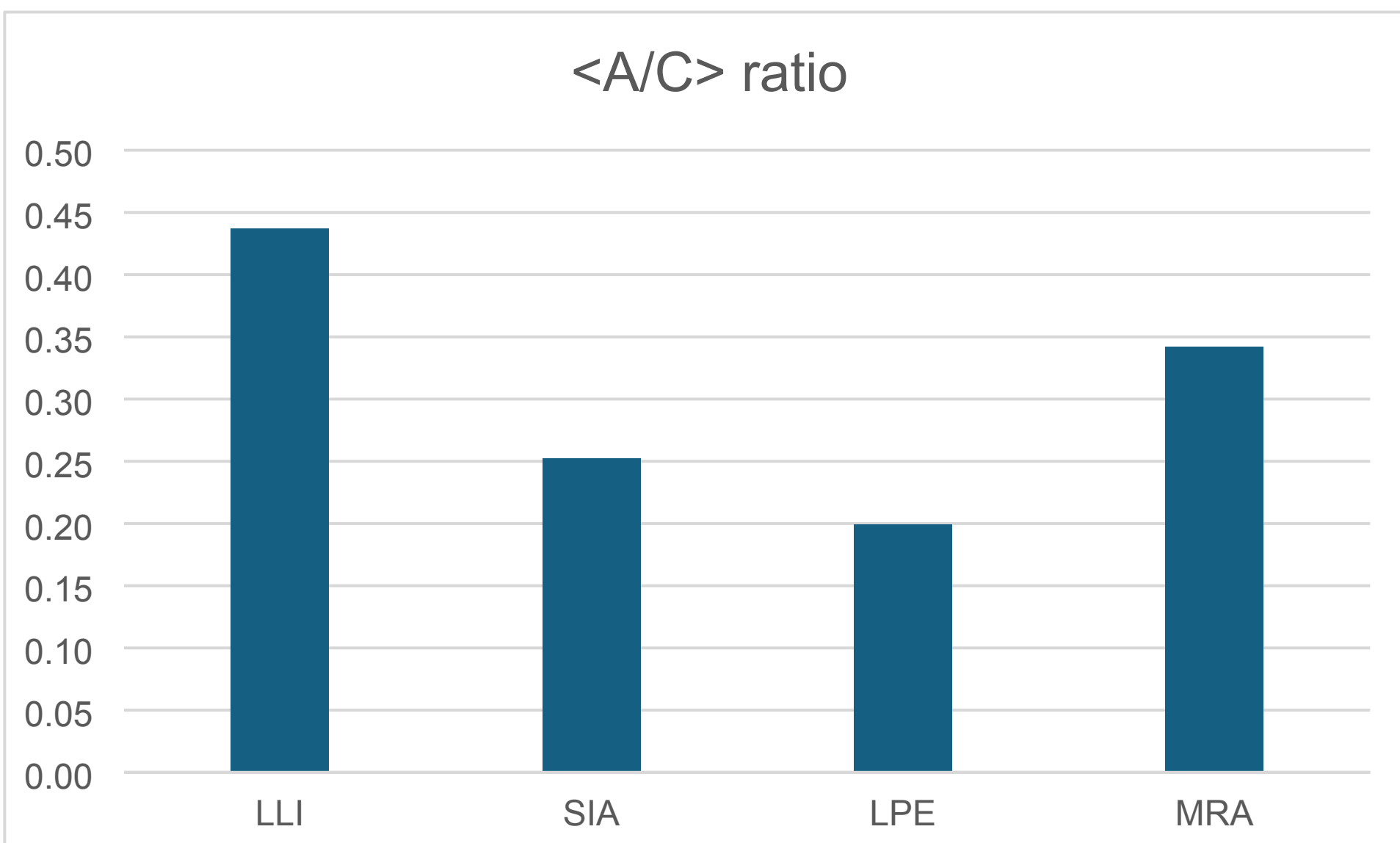

Figure S5. Histogram summarizing the amorphous/crystalline (A/C = A_{950}/A_{960}) ratios for the four analyzed samples.

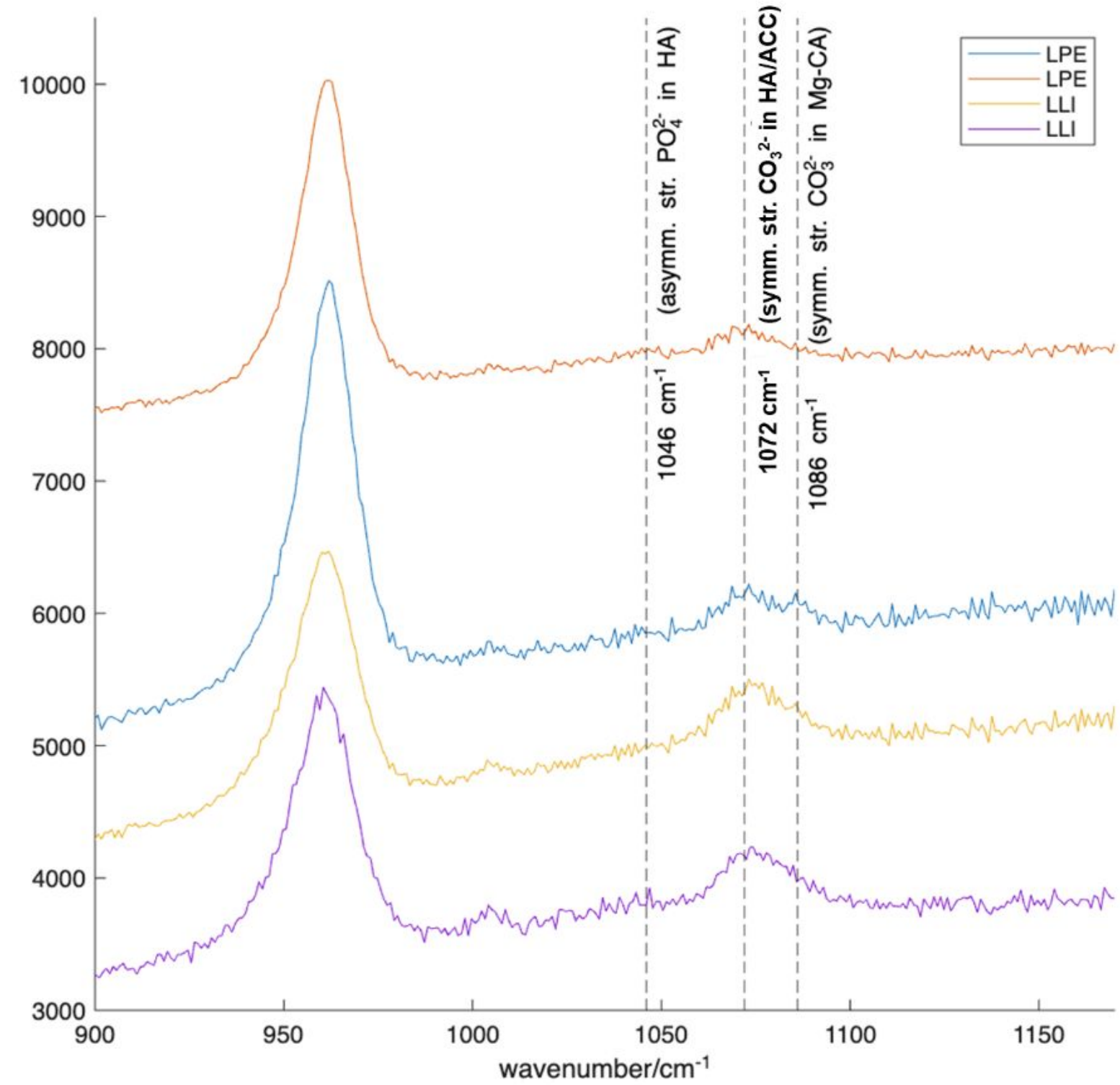

Figure S6. Variations of the Raman signals in the carbonate stretching region for selected samples.

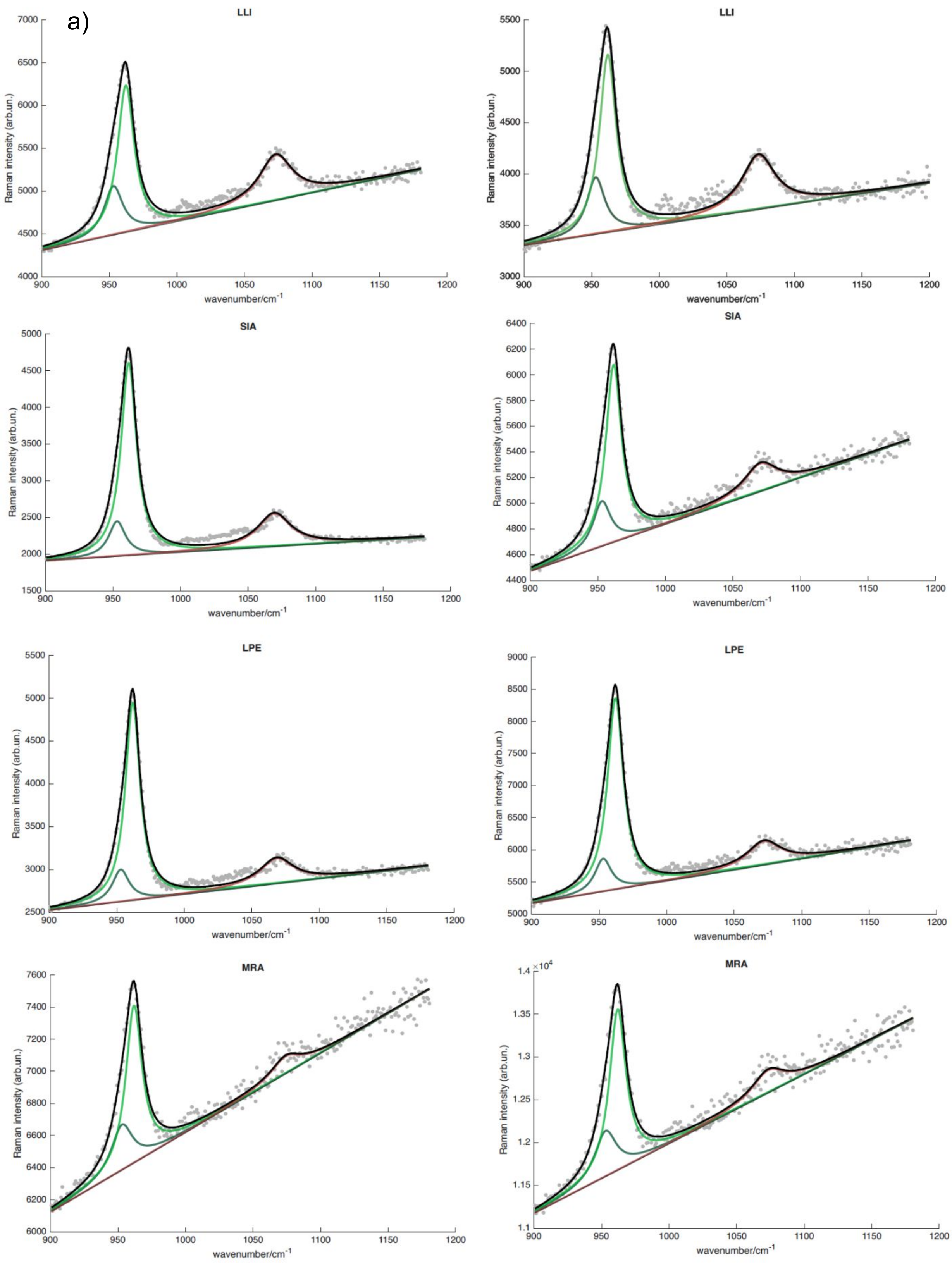
a)
LLI
LLI
SIA
SIA
LPE
LPE
MRA
MRA
Raman intensity (arb.un.)
wavenumber/cm-1
×10^4

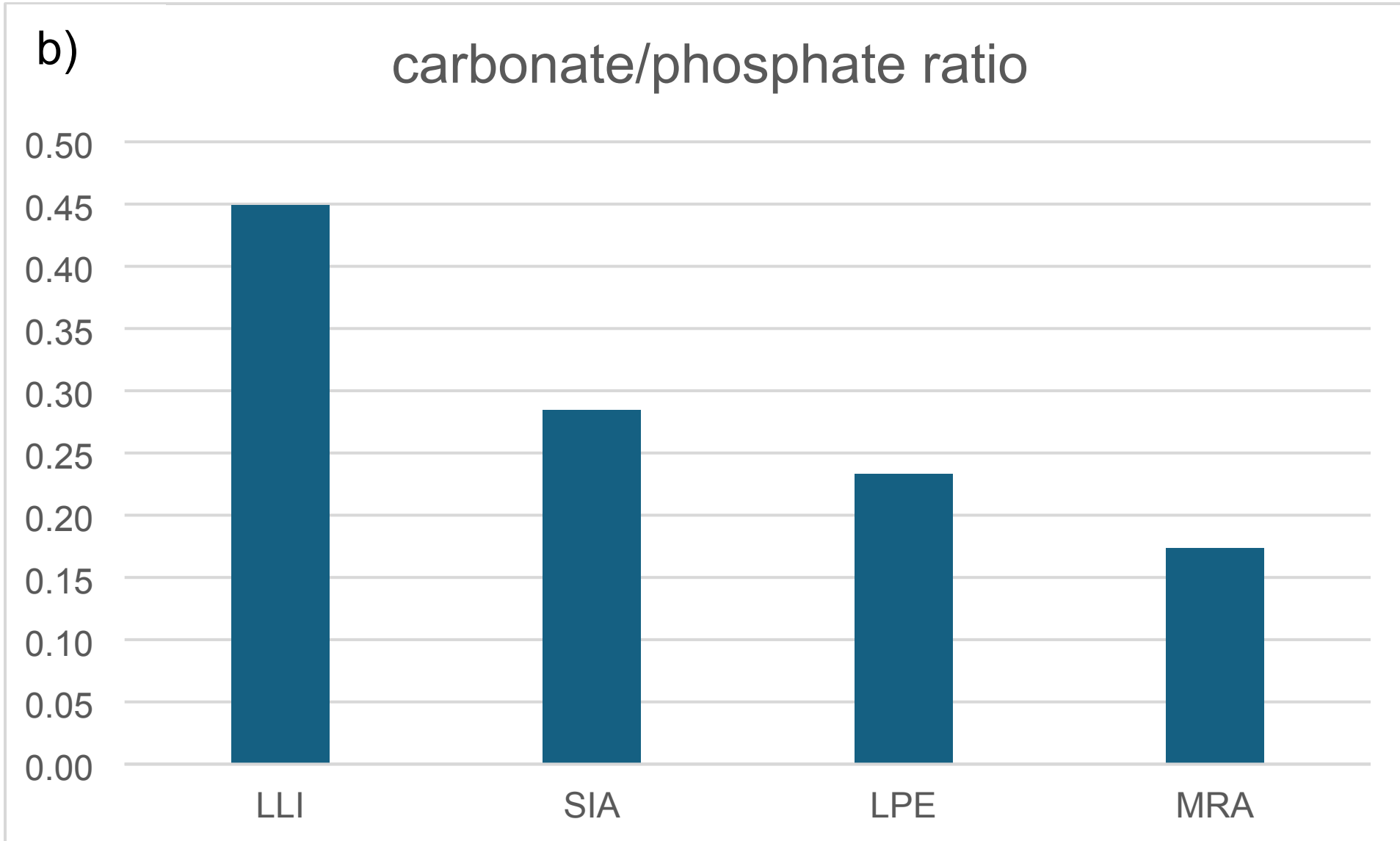

Figure S7. a) Spectral deconvolution in the 900–1200 cm^{-1} region of the Raman spectra (two regions for each sample) of the LLI, SIA, LPE, and MRA samples. The deconvolution used a linear background and three Lorentzian lineshapes (see text for details). b) Trend of the carbonate/phosphate Raman intensity ratios for the four samples.

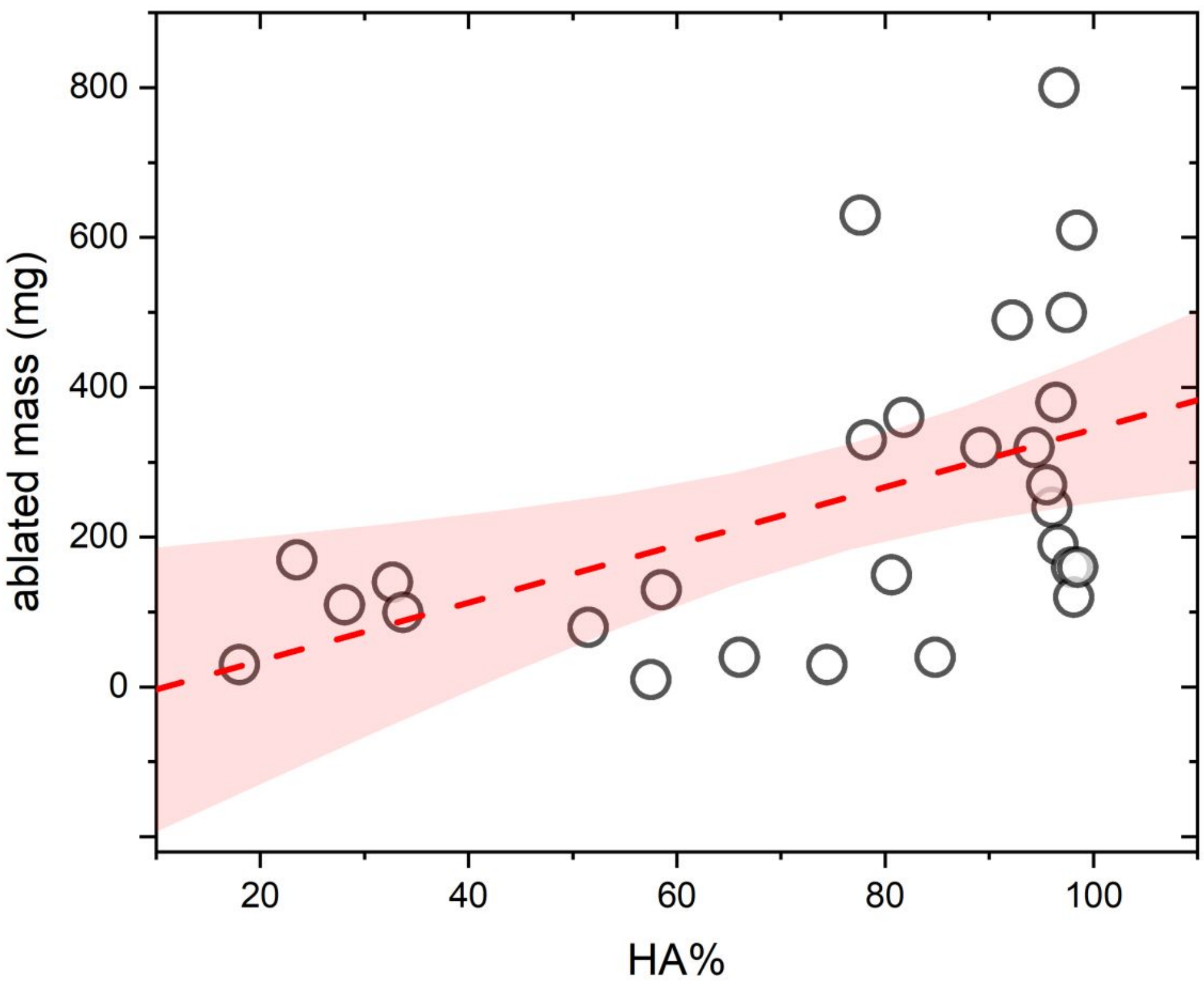

Figure S8. Ablated calculus mass as a function of HA%. The red dashed line represents the linear regression of the data, giving a mass increment per HA% of (3.9 ± 1.3) mg. The pink area represents the 95% confidence band.

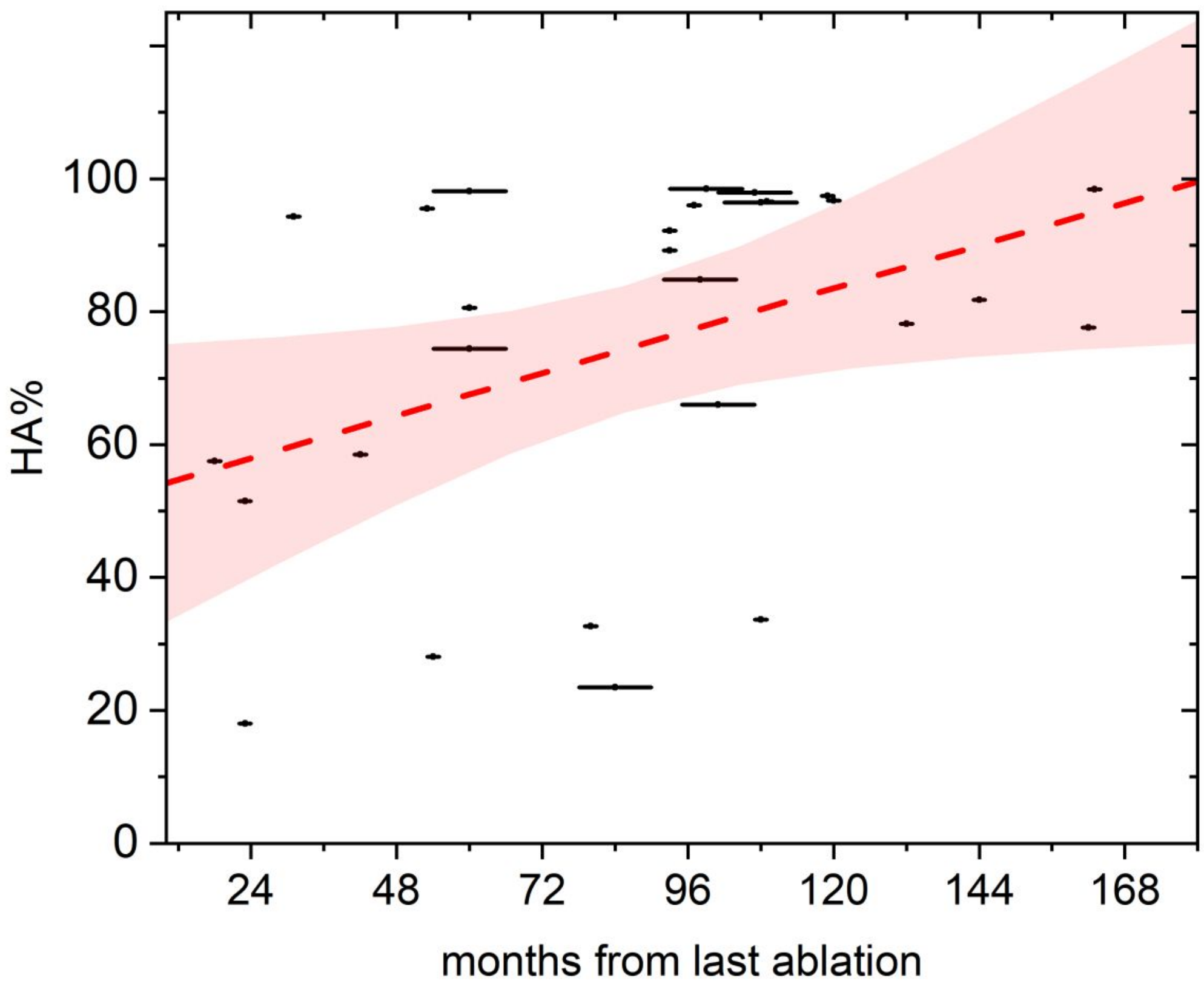

Figure S9. Weight percent of HA as a function of months from last ablation. The thick red dashed line represents the linear regression of the data, giving (0.27 ± 0.12)%/mo). The pink area represents the 95% confidence band.

Table S5. Exploratory multiple linear regression models using the XRPD-derived crystalline hydroxyapatite fraction (HA%) as dependent variable.

Table S5a. Summary of multiple linear regression models.

Model	Dataset	Dependent variable	Predictors	n	R^2	Adjusted R^2	F statistic	Model p-value	VIF range	Interpretation
**M1**	Complete dataset	HA%	Age at ablation, time elapsed since previous dental cleaning, ablated calculus mass	28	0.287	0.198	F(3,24) = 3.22	0.0405	1.76–3.48	The model is statistically significant, but the explained variance is moderate. None of the individual predictors remains significant when considered simultaneously, indicating partial covariation among age-, accumulation- and mass-related descriptors.
**M2**	Subset with available oral-health score	HA%	Age at ablation, time elapsed since previous dental cleaning, ablated calculus mass, oral-health score	23	0.307	0.153	F(4,18) = 1.99	0.139	1.04–4.47	Addition of the exploratory oral-health score slightly increases R^2 but does not yield a statistically significant model. The oral-health score should therefore be regarded as descriptive rather than predictive.

Table S5b. Regression coefficients.

Model	Predictor	Coefficient	Standard error	t statistic	p-value	VIF
**M1**	Intercept	47.11	12.79	3.68	0.00117	—
**M1**	Age at ablation (months)	0.204	0.213	0.96	0.347	3.17
**M1**	Time elapsed since previous dental cleaning (months)	−0.0468	0.211	−0.22	0.826	3.48
**M1**	Ablated calculus mass (mg)	0.0472	0.0294	1.61	0.121	1.76
**M2**	Intercept	45.02	18.46	2.44	0.0253	—
**M2**	Age at ablation (months)	0.0355	0.320	0.11	0.913	4.47
**M2**	Time elapsed since previous dental cleaning (months)	0.0617	0.284	0.22	0.831	4.34
**M2**	Ablated calculus mass (mg)	0.0553	0.0327	1.69	0.108	1.73
**M2**	Oral-health score	3.49	5.60	0.62	0.541	1.04

Note: HA% refers to the semi-quantitative hydroxyapatite fraction within the crystalline fraction resolved by XRPD. The oral-health score was used as an exploratory ordinal descriptor derived from available veterinary records and should not be interpreted as formal periodontal staging. VIF: variance inflation factor.

Table S6. Principal component analysis of age-, accumulation-, mineralogical- and chemical descriptors.

Table S6a. PCA performed on the four core quantitative variables available for all dogs.

Principal component	Eigenvalue	Explained variance (%)	Cumulative variance (%)	Main interpretation
**PC1**	2.735	68.4	68.4	Common age–accumulation–mineral maturation axis, with positive contributions from age, time elapsed since previous dental cleaning, ablated mass and HA%.
**PC2**	0.679	17.0	85.4	Deviation from the age-accumulation-composition axis, mainly separating HA-rich samples from samples characterized by relatively higher age or accumulation descriptors but lower HA%.
**PC3**	0.416	10.4	95.8	Secondary residual variability, mainly associated with ablated mass.
**PC4**	0.170	4.2	100.0	Minor residual component.

Table S6b. PCA loadings for the four core quantitative variables.

Variable	PC1	PC2	PC3	PC4
**Age at ablation (months)**	0.888	−0.263	0.258	−0.277
**Time elapsed since previous dental cleaning (months)**	0.890	−0.326	0.113	0.298
**Ablated calculus mass (mg)**	0.832	0.051	−0.549	−0.055
**HA%**	0.680	0.708	0.188	0.038

Table S6c. PCA performed on the subset of samples with complete chemical data.

Principal component	Eigenvalue	Explained variance (%)	Cumulative variance (%)	Main interpretation
**PC1**	3.195	35.5	35.5	Mixed maturation/composition axis, with positive contributions from HA%, age, accumulation descriptors, mass and Mg%, and negative contributions from 100/HA% and Ca/P.
**PC2**	2.297	25.5	61.0	Chemical contrast axis, dominated by positive Ca/P and 100/HA% loadings and negative P% and HA% loadings.
**PC3**	1.215	13.5	74.5	Secondary chemical variability, mainly contrasting Ca% with P% and age-related descriptors.
**PC4**	1.018	11.3	85.8	Residual variability not directly attributable to the main maturation/composition trend.
**PC5**	0.570	6.3	92.1	Minor residual component.

Table S6d. PCA loadings for the subset with complete chemical data.

Variable	PC1	PC2	PC3
**HA%**	0.832	−0.393	−0.169
**100/HA%**	−0.758	0.567	0.180
**Age at ablation (months)**	0.534	0.617	0.434
**Time elapsed since previous dental cleaning (months)**	0.632	0.555	0.342
**Ablated calculus mass (mg)**	0.741	0.363	0.102
**Ca%**	0.278	0.192	−0.670
**Mg%**	0.646	0.206	−0.317
**P%**	0.034	−0.515	0.453
**Ca/P**	−0.446	0.811	−0.290

Note: PCA was performed on standardized variables. Loadings are reported as correlations between variables and principal components. HA% refers to the semi-quantitative hydroxyapatite fraction within the crystalline fraction resolved by XRPD. The PCA was used as an exploratory tool to identify axes of covariation and not as a predictive or diagnostic model.

References

(1) Hanawalt JD, Rinn HW and Frevel LK (1938) Chemical Analysis by X-Ray Diffraction. Anal. Chem., 10, 475-512.

(2) Paquette J, Reeder JR (1990) Single crystal X-ray structure refinement of two biogenic magnesian calcite crystals. Am. Mineral., 75, 1151-1158.

(3) Falini G, Fermani S, Gazzano M, Ripamonti A (1998) Structure and morphology of synthetic magnesium calcite. J. Mater. Chem., 8(4), 1061–1065, https://doi.org/10.1039/a707893e

(4) Fahad M & Saeed S (2018) Determination and estimation of magnesium content in the single phase magnesium-calcite [$Ca_{(1-x)}Mg_xCO_{3(s)}$] using electron probe micro-analysis (EPMA) and X-ray diffraction (XRD) Geosci. J., 22(2), 303-312, http://dx.doi.org/10.1007/s12303-017-0059-8

(5) Goldsmith JR, Graf DL, Joensuu OI (1955) The occurrence of magnesian calcites in nature, Geoch. Cosmoch. Acta, 7(5–6), 212-230, https://doi.org/10.1016/0016-7037(55)90033-8

(6) Rietveld HM (1969) A profile refinement method for nuclear and magnetic structures. J. Appl. Crystallogr. 2, 65-71, https://doi.org/10.1107/ S0021889869006558

(7) Döbelin N & Kleeberg R (2015) Profex: a graphical user interface for the Rietveld refinement program BGMN. J. Appl. Crystallogr., 48(5), 1573-1580, https://doi.org/10.1107/S1600576715014685
